# The Interplay of Chronic Stress and Cancer: Pathophysiology and Implications for Integrated Care

**DOI:** 10.1002/cnr2.70143

**Published:** 2025-05-19

**Authors:** Joyeeta Talukdar, Megha, Hemant Choudhary, Sushma Bhatnagar, Anuja Pandit, Ashwani Kumar Mishra, Subhradip Karmakar, Pratap Sharan

**Affiliations:** ^1^ Department of Bio‐Chemistry All India Institute of Medical Sciences New Delhi India; ^2^ Department of Psychiatry All India Institute of Medical Sciences New Delhi India; ^3^ Department of Onco‐Anaesthesia & Palliative Medicine DR. B.R.A.I.R.C.H, All India Institute of Medical Sciences New Delhi India; ^4^ National Drug Dependence Treatment Centre All India Institute of Medical Sciences New Delhi India

**Keywords:** cancer, cancer stem cells, chronic stress, counselling, depression, immune modulation, palliative care, psychiatry, psycho‐oncology, psychotherapy

## Abstract

**Background:**

Cancer‐associated depression is a multifaceted condition that arises from the interplay of biological, psychological, and social factors in individuals diagnosed with cancer. Understanding this condition involves exploring how cancer and its treatments can precipitate depressive symptoms and the mechanisms behind this association. Chronic stress, inflammation, and immunological responses play a crucial role in the development of both cancer and depression. The objective of this review is to describe and synthesize information on the complex interactions between chronic stress, inflammation, immunological responses, and cancer development. Additionally, it aims to review existing evidence regarding mechanisms such as neurotransmitter imbalances, structural brain changes, and genetic predispositions as key contributors to depression in cancer patients.

**Recent Findings:**

A comprehensive literature search on Cancer‐associated Depression was conducted in electronic databases, including APA PsycINFO, Medline, Google Scholar, Embase, PubMed, Scopus, and Web of Science. The research focused on understanding the potential relationship between stress‐induced depression and cancer by examining neurochemical, anatomical, immunological, genetic, and psychological changes. The findings revealed a compilation of both quantitative and qualitative studies on depression in cancer patients. Evidence suggested a potential link between cancer‐induced stress and depression, with increased levels of proinflammatory cytokines (such as IL‐6) and dysregulation of neurotransmitters, including serotonin, contributing to the onset of depression. Furthermore, studies indicated that antidepressants, along with psychological interventions, were effective in managing depression among cancer patients.

**Conclusion:**

This narrative review provides insights into the importance of integrating oncology and mental health services to address the psychosocial needs of cancer patients. Future research should focus on the bidirectional interactions between stress and cancer, aiming to improve cancer care by incorporating mental health support. Addressing the mental health aspects of cancer treatment can significantly enhance patient outcomes and overall quality of life.

## 1 Introduction

### 1.1 Background and Significance

Depression, recognized as a pervasive and severe mood disorder, manifests through persistent sadness, hopelessness, and disinterest, adversely affecting one's daily life. Its complexity lies in the amalgamation of genetic, environmental, neurochemical, and physiological elements. Scientific studies have pinpointed neurotransmitter imbalances involving serotonin, norepinephrine, and dopamine, structural alterations in the brain like hippocampal shrinkage and modifications in the prefrontal cortex, and genetic factors highlighting the disorder's multifaceted genetic‐environment interplay. The initial adaptive response to stressful stimuli involves the immune system, the hypothalamic–pituitary–adrenal (HPA) axis, and the sympathetic‐adreno‐medullar (SAM) system activation [1]. Therefore, in reaction to real or imagined risks to homeostasis, the brain circuitry of the hippocampus, amygdala, and prefrontal cortex processes information about the stressor and triggers physiological mechanisms of adaptation mediated primarily by catecholamines and glucocorticoids [2]. By controlling glucocorticoid secretion, the HPA axis, in addition to the autonomic nervous system, is a major component of the stress response. The hypothalamic release of corticotropin‐releasing hormone (CRH) is a characteristic of this HPA‐mediated stress response. CRH acts on the anterior pituitary to stimulate the secretion of adrenocorticotropic hormone (ACTH), which in turn causes the adrenals to secrete cortisol (humans) or corticosterone (rodents). When the HPA axis and SAM system are activated together, more glucose is broken down and energy is redistributed throughout a variety of tissues and organs, including the brain [1]. According to recent research, different brain areas regulate the immune system by driving a particular leukocyte trafficking pattern during periods of acute psychological stress [3]. The degree and duration of the stressor significantly impact the modification of immune function due to stress [4].

These insights from comprehensive research illustrate depression as a disorder influenced by a spectrum of neurochemical, genetic, and inflammatory factors [5, 6, 7, 8, 9, 10, 11]. The correlation between depression and cancer is significant, with heightened instances of depression and anxiety noted among cancer patients. Chronic stress exposure frequently results in severe immunosuppression, a reduction in the number and function of immune cells, and an imbalance in the ratio of Type 1 to Type 2 cytokines. Immune malfunction brought on by stress has a role in the persistent low‐grade inflammation that is intimately linked to common chronic illnesses like cancer. However, cancer leads to an inflammatory environment that manifests systemically [12], which modifies the activation of the HPA axis and the proper secretion of cortisol in response to stress [13]. The perception that depression is a normal and universal reaction to serious illnesses has led to the underdiagnosis and undertreatment of depression in cancer patients complicating treatment, recovery, and overall quality of life [14, 15, 16, 17, 18, 19, 20].

### 1.2 Scope of the Review

This review delves into the intricate dynamics between chronic stress, inflammation, and cancer, highlighting shared pathways that underlie these conditions. Chronic stress and cancer induce a systemic pro‐inflammatory state through common signaling pathways such as NF‐kB, STAT3, and mTOR, promoting tumor growth and metastasis while also contributing to neuroinflammation [21]. The reciprocal relationship between stress and cancer is evident in their mutual promotion of neuroinflammation, involving monocyte trafficking, microglial activation, and blood–brain barrier disruptions. Cancer stem cells (CSCs) are a subset of cancer cells capable of driving tumor growth, metastasis, and therapeutic resistance [22, 23, 24]. Emerging evidence suggests a complex interplay between chronic psychological stress, neuroinflammation, and the maintenance of CSC stemness [25, 26, 27]. This relationship may contribute to cancer progression, emphasizing the importance of addressing both physical and mental health aspects in cancer care.

Addressing the mental well‐being of cancer patients therefore is a pressing global healthcare challenge. This underscores the importance of a holistic approach to cancer care, integrating mental health considerations. As attention shifts toward cancer recurrence surveillance, concerns arise regarding potential oversight of long‐term effects on both mental and physical health [28]. This review emphasizes the necessity for further research to understand the bi‐directional relationship between stress and cancer, aiming to enhance cancer care by integrating mental health support. It delves into survivors mental health challenges, offering solutions and insights into areas for future research, like precision medicine's potential for stress‐reducing cancer treatments. Additionally, it identifies gaps in cancer care and anticipates future advancements in tailored, holistic care, boosting emotional and physical well‐being, and increasing survivor quality of life.

## 2 Understanding Depression in Cancer Patients

### 2.1 Prevalence and Impact

Stress, a ubiquitous aspect of human experience, arises from a complex interplay of psychological, mental, physical, and emotional factors. Individuals are exposed to stress from daily responsibilities, occupational demands, interpersonal relationships, and external circumstances such as adverse childhood experiences, environmental toxins, socioeconomic disparities, and discrimination, all of which can significantly impact their overall well‐being [29]. Further, chronic stress characterized by prolonged exposure to stressors and associated allostatic load (AL) has been shown to have deleterious effects on health, including increased risk of insomnia, gastrointestinal disorders, anxiety, depression, and cardiovascular disease [30, 31, 32, 33, 34, 35]. Furthermore, they can disrupt the balance of the neuroendocrine‐immune system, contributing to the development and progression of cancer [36, 37, 38]. For example, using AL as a stress marker, recent studies have found that women with high AL had a 64% increased risk of cancer [39].

### 2.2 Factors Contributing to Depression in Cancer

Depression in cancer patients is an intricate and multifaceted issue that is influenced by various factors. Such individuals diagnosed with cancer have a high probability of suffering from anxiety and depression, which comes from the interaction of numerous biological, psychological, and social factors [40] (Summarized in Table 1).

**TABLE 1:** Factors that may contribute to depression among cancer patients [40].

Individual characteristics	Psychological factors	Social factors	Cancer‐specific factors
Age Gender Ethnicity Sexuality Religion Disability Marital status	Emotional distress Coping behavior Denial Anger Fear Grief Resilience Sensitivity to others Self/body Image issues	Educational level Employment status Type of occupation Household income and wealth Family Social support Belief system Healthcare system Access to care Welfare system	Type of cancer Diagnosis experience Symptoms Stage Grade Prognosis Curability Recurrence Functional deterioration Impairment

Biological factors	Prior psychological factors	Lifestyle factors	Treatment factors
Genetic susceptibility Neurochemical changes Hormonal changes (e.g., cortisol) Chronic pain Immune system functioning Co‐morbid health conditions (e.g., Infections, neurological disorders, cardiac and respiratory disorders)	Personality (e.g., Type C, Type D, neuroticism) History of psychiatric disorder Previous suicidal behavior Prior traumatic event/abuse	Consumption of tabacco, alcohol, drugs Diet Obesity Physical activity Sleep Stress	Setting (inpatient, outpatient) Length and burden Phase (e.g., acute, palliative) Treatment modality (e.g., surgery, chemotherapy, radiotherapy) Dose Side effects Long‐term complications (fatigue, cognitive changes, secondary cancers) Cost of treatment Response to treatment

#### 2.2.1 Individual Factors

An array of characteristics such as age, gender, ethnicity, sexuality, religion, disability, and marital status, possibly play a role in the development of depression in this population [40]. As age is a major predictor, elderly people are more susceptible to depressive symptoms owing to various factors like comorbidities and physical health decline [41]. Conversely, younger patients often experience a high level of psychological distress as their life planning and career events are disrupted [42]. Research into gender differences indicates that women are more likely to suffer depression after cancer diagnosis compared with men, due to biological influences including hormonal changes as well as differing coping mechanisms [43]. For men, however, the underreporting of emotional distress might lead to an underestimation of the prevalence of depression in this population [44]. Ethnic studies have shown that minority groups often face barriers due to stigma or systemic inequalities [45]. Religion has been shown to provide a level of solace, but also to imply guilt and conflict regarding illness about religious beliefs [46]. Sexuality is also a significant variable, as cancer survivors who are LGBTQ+ may experience heightened psychological distress as a result of stigma or lack of targeted healthcare services [47]. Disabilities can make physical and psychological challenges worse in cancer, reducing self‐efficacy and fostering feelings of isolation [48]. In addition, marriage has a significant impact on mental health; married persons generally record fewer depressive symptoms owing to the intimacy of their marriage, while single or widowed patients can exhibit higher levels of loneliness and isolation [49]. These interrelated variables indicate a need for culturally adapted interventions in comprehensive cancer care.

#### 2.2.2 Biological Factors

Biological factors contribute to depression through inflammation, HPA axis, glutamate excitotoxicity, and genetic predisposition like 5‐HTTLPR and MAOA genes [50]. Other genetic factors like 5‐HTTLPR and MAOA polymorphism increase the risk of depression by altering the serotonin levels and stress response pathways [51, 52]. Neurochemical imbalances are known to be the main cause of depression, which includes low levels of serotonin, norepinephrine, and dopamine [5, 6, 53]. Hormonal changes especially those caused by cancer treatments like endocrine therapies for breast or prostate cancer are also significant contributors to depression. These therapies affect the levels of estrogen, testosterone, and cortisol which disrupt the neurochemical balance and leads to mood dysregulation [54]. Elevated proinflammatory cytokines like IL‐6 and TNF‐α are associated with depression in cancer patients [8, 55]. Cancer‐related pain is strongly associated with depression and emotional distress [56].

#### 2.2.3 Psychological Factors

Research studies show emotional distress, including anger, fear, grief, and body image issues as highly relevant contributors to depression in cancer patients [57, 58]. Many studies have showed that higher levels of distress, notably anger and fear, were associated with elevated symptoms of depression among cancer patients [40, 59]. The studies also showed that problems relating to body image were correlated with a higher incidence of depression among women with breast cancer [60]. Grieving and loss, in studies, have been associated intensely with the mental health of cancer patients, and it has been suggested that unresolved grief can heighten an individual's risk for depression [61]. Factors such as coping behaviors, resilience, sensitivity to others, and self‐image have a huge impact on the development of depression among patients [62, 63, 64, 65]. Bad forms of coping like avoidance and denial can serve to exacerbate distress, while good types like problem‐focused coping can help diminish depressive symptoms [66]. Resilience, or the ability to bounce back from adversity, varies in extent among individual patients and is negatively correlated with the severity of depression [67]. Greater sensitivity to others, including fear of burdening loved ones, can magnify feelings of guilt and loneliness [49]. Moreover, changing self‐image because of a physical altercation, for example, loss of hair or disfigurement can also have secondary effects on emotional well‐being and depressive symptoms [68].

#### 2.2.4 Prior Psychological Factors

Personality traits such as Type C (characterized by suppression of emotions and excessive agreeableness) and Type D (distress‐prone) are associated with higher psychological distress and poorer coping mechanisms [42, 69, 70]. Neuroticism, marked by emotional instability, predisposes individuals to heightened vulnerability to stress and negative affect [71]. A history of psychiatric illness or previous suicidal behavior further increases the risk of depression, as these individuals often have impaired emotional resilience [17, 72, 73, 74]. Moreover, prior traumatic events or abuse can lead to chronic stress responses and maladaptive coping, making such individuals more susceptible to depression during the cancer journey [75, 76]. These factors highlight the necessity of comprehensive psychological screening in cancer care.

#### 2.2.5 Social Factors

Social factors significantly contribute to the development and severity of depression in individuals with cancer, underscoring the complex interplay between social determinants of health and mental well‐being. Educational level often influences health literacy, impacting a patient's understanding of their diagnosis and treatment, which may heighten distress [77, 78]. Employment status and type of occupation are critical, as job loss or inability to work due to cancer can lead to financial strain and reduced self‐esteem, exacerbating depressive symptoms [79, 80]. Similarly, household income and access to welfare systems determine the availability of resources for coping with illness, while inadequate financial means increase vulnerability to psychological distress [81]. Family type and social support are pivotal; individuals with supportive family structures report lower depression levels compared to those in isolated or strained relationships [82, 83]. Cultural belief systems and access to care also play a role, influencing attitudes toward treatment and the likelihood of seeking psychological help [84]. Together, these factors create a multifaceted framework that shapes the mental health outcomes of cancer patients.

Socioeconomic status (SES) further compounds the risk of depression among cancer patients. Low SES, often characterized by unemployment, limited educational attainment, and financial strain, can intensify stress levels, reduce access to healthcare, and hinder the ability to seek adequate psychosocial support [85]. Social support, or the lack thereof, is a critical protective factor. Patients with limited social networks or those experiencing social isolation are more susceptible to feelings of hopelessness and depression [86].

#### 2.2.6 Lifestyle Factor

Depression risk in cancer patients may be attributed to a variety of lifestyle and environmental factors, such as substance abuse, diet, obesity, exercise, sleep, and stress. Cancer patients consuming tobacco, alcohol, and drugs remain at an elevated risk of mood disorders such as depression [87, 88]. Such substances can worsen psychological distress, render inadequate coping strategies, and compromise treatment outcomes. A poor diet with a deficit of dietary nutrients creates adverse effects on brain chemistry and makes individuals more vulnerable to depression through their interference with metabolic and inflammatory pathways [89, 90]. Similarly, obesity also promotes increased levels of inflammation and creates hormonal imbalances, affecting mood regulation and causing depression [91, 92]. Besides, insufficient physical activity might worsen the development of depression by limiting the benefits of exercise, which might produce endorphins and serotonin contributing to mood regulation [93]. Sleep disturbances in cancer patients may also serve as a significant risk factor since poor sleep quality and quantity are strongly associated with more severe depressive symptoms [94, 95]. Finally, chronic stress in cancer, especially upon diagnosis and treatment, plays a vital role in depression by altering physiology within the brain that amplifies emotional distress [96].

#### 2.2.7 Cancer‐Specific Factors

Among cancer‐related factors, those related to specific characteristics of cancer make a profound impact on depression development because cancer affects the physical, emotional, and social aspects. The type of cancer can have an influence on mental well‐being as certain cancers like the brain or pancreas have a more unfavorable prognosis and generate profound psychological distress [97]. The diagnosis of cancer experience itself evokes fear, uncertainty, stigma, and acute stress, often resulting in depression [98]. Research studies show Permanent pain, fatigue, or deformity worsens the state of distress [40]. The stage and grade of a cancer are associated with the severity of depression, more specifically the level of hopefulness that it provides and its mental health outcome [99]. Similarly, prognosis and curability influence the patient's outlook on life; a poor prognosis may foster hopelessness [94]. Recurrence stops being another tear in an emotional curtain and, instead, manifests itself in fear and psychological distress [100]. Functional decline together with imposed physical limitations owing to cancer or treatment could lead to a loss of independence and quality of life, thus exacerbating depression [101]. An understanding of this multifaceted set of factors and their relation to depression is critical for a holistic approach to cancer patients.

#### 2.2.8 Treatment Factors

The nature of the cancer is a key determinant, with certain cancers associated with poorer prognosis, including lung and pancreatic cancers, being more strongly correlated with higher levels of depression [102]. In addition, the treatment setting‐inpatient versus outpatient‐might influence psychological well‐being. Inpatient settings, in general, are often associated with more intensive care, leading to possible increased feelings of isolation and anxiety [103]. Hence, the duration of treatment and the burden of treatment may also lead to depression since long therapies may cause chronic distress than effective treatments due to their toll, both physically and emotionally [83]. Experimental studies indicate that longer treatment intervention with either chemotherapy or radiotherapy is associated with other possible factors associated with increased risk for depression, especially when side effects like fatigue, cognitive impairment, and nausea arise [104]. The phase of the disease is an important factor, as such patients may experience more emotional distress in acute or palliative phases due to uncertainties concerning prognosis and severity of symptoms [98]. The financial burden of cancer treatment also links with depression through the added economic strain consequent to high treatment costs and subsequent feelings of helplessness and anxiety [105]. Finally, the treatment outcome represents an important variable. Poor behavioral outcomes following chemotherapy were associated with severe depression, as patients who responded poorly to cancer therapies exhibited significantly higher depression scores compared to those with positive therapeutic outcomes [106].

### 2.3 Psychobiological Underpinnings of Depression in Cancer Patients

Depression in cancer patients is a complex phenomenon influenced by various psychobiological factors. Understanding the neurobiological, immunological, genetic, and epigenetic aspects of depression in this population is crucial for developing effective interventions. This section explores the psychobiological underpinnings of depression in cancer patients, focusing on neurobiological factors such as neurotransmitter dysregulation and HPA axis dysfunction, immunological factors including inflammatory responses and immune system modulation, as well as genetic and epigenetic influences.

#### 2.3.1 Neurobiological Factors

##### 2.3.1.1 Neurotransmitter Dysregulation

Neurotransmitter dysregulation, particularly involving serotonin, norepinephrine, and dopamine, plays a significant role in the development of depression in cancer patients. Serotonin imbalance, often referred to as the “feel‐good” neurotransmitter, is a well‐documented contributor to depression [5]. Similarly, alterations in norepinephrine and dopamine levels have been implicated in mood disorders [6]. Under chronic stress, region‐specific neuronal remodeling occurs in brain areas such as the hippocampus, amygdala, and prefrontal cortex [107]. Chronic stress leads to sustained synaptic plasticity in the prefrontal cortex and induces varying dendritic changes in the amygdala and hippocampal neurons [108]. Dysregulation of these neurotransmitters disrupts mood regulation and emotional processing, contributing to depressive symptoms among cancer patients.

##### 2.3.1.2 HPA Axis Dysfunction

Repeated exposure to stressors increases hypothalamic CRH gene and protein expression, enhancing cellular excitability by increasing the density of catecholaminergic and glutamatergic terminals on CRH neurons. This leads to chronic HPA axis activation, resulting in glucocorticoid hypersecretion and sensitized stress responses [109]. In addition to prolonged HPA axis activation, chronic stress alters locus coeruleus‐norepinephrine function, with growing evidence suggesting that increased sympathetic activity also contributes to glucocorticoid hypersecretion following chronic stress [110]. Chronic stress associated with cancer diagnosis and treatment can lead to dysregulation of the HPA axis, resulting in elevated cortisol levels [9]. High cortisol levels have been linked to depressive symptoms and impaired mood regulation in cancer patients, highlighting the role of HPA axis dysfunction in depression pathogenesis.

#### 2.3.2 Immunological Factors

##### 2.3.2.1 Inflammatory Responses

Inflammatory responses, characterized by elevated levels of proinflammatory cytokines such as interleukin‐6 (IL‐6) and tumor necrosis factor‐alpha (TNF‐α), have been implicated in the development of depression in cancer patients [8]. It is widely acknowledged that both Androgen Deprivation Therapy (ADT) and second‐generation anti‐androgens, used to treat prostate cancer, are linked to a higher risk of depression and anxiety [111, 112, 113]. In a landmark study encompassing 37 388 prostate cancer patients undergoing ADT, 10.6% were diagnosed with depression or anxiety [112]. Despite these findings, recent studies have shown that an elevated level of IL‐6, related to ADT treatment for prostate cancer, is associated with increased fatigue but not with depressive symptoms [114]. This evidence supports the idea that cancer patients experiencing fatigue, anxiety, and depression may have elevated levels of circulating inflammation markers, such as interleukin‐6 (IL‐6) and C‐reactive protein (CRP) [115, 116, 117, 118]. Furthermore, administering cytokines, which are involved in triggering the inflammatory response, can lead to symptoms of fatigue and depression in both humans and animals [119]. Clinical studies have revealed that roughly 30% to 45% of patients undergoing IFN‐α therapy experience depression during treatment including that of cancer [120, 121]. A double‐blind, placebo‐controlled study of patients undergoing IFN‐α therapy for malignant melanoma demonstrated that pretreatment with the antidepressant paroxetine was effective in preventing the development of major depression and was linked to improved adherence to IFN‐α therapy [120]. Chronic inflammation, triggered by cancer‐related factors and treatment modalities, can hence disrupt neurobiological pathways involved in mood regulation and contribute to depressive symptoms.

The occurrence of depression in patients with glioma and the ability of inflammatory cytokines to predict it was investigated by Li et al. Among 203 patients with glioma, 66.5% showed depressive symptoms. Proinflammatory cytokines, including interleukin (IL)‐6 and tumor necrosis factor (TNF)‐α, demonstrated good performance in accurately predicting depression in these patients [122]. These inflammatory cytokines have potential to be effective clinical screening and diagnostic tools, as well as biomarkers for depression in patients with glioma. Another study examined the prediction ability of various circulating cytokines for depression in patients with breast cancer not receiving adjuvant chemotherapy. The researchers found that the proinflammatory cytokine IL‐2 and the anti‐inflammatory cytokine IL‐5 demonstrated good predictability for depression, even after controlling for covariates. IL‐2 had the best prediction ability among the cytokines studied, with a sensitivity of 86.7% and a specificity of 52.9% at an optimal cut‐off value of 1.06 pg/mL. The findings suggest that circulating cytokines may be a valid laboratory diagnostic tool for depression in cancer patients [123].

Moreover, inflammation‐induced alterations in neurotransmitter metabolism and synaptic plasticity may further exacerbate depression in this population [124].

##### 2.3.2.2 Immune System Modulation

Immune system modulation, including changes in immune cell function and activity, has been associated with depression in cancer patients. Dysregulation of immune responses, characterized by altered cytokine profiles and immune cell activation, may contribute to the pathophysiology of depression in this population [124]. The neuroendocrine system, particularly the hypothalamic–pituitary–adrenal (HPA) axis, plays a crucial role in the connection between immune dysregulation and depression in cancer patients [96, 125, 126].

The HPA axis is a key component of the stress response system. When the body encounters stressors, the hypothalamus releases corticotropin‐releasing hormone (CRH), which stimulates the pituitary gland to secrete adrenocorticotropic hormone (ACTH). ACTH then triggers the adrenal glands to produce glucocorticoids, such as cortisol, which help the body respond to stress [127]. In cancer patients, pro‐inflammatory cytokines released by the tumor or as a result of cancer treatments can overstimulate the HPA axis, leading to chronic hyperactivity [128, 129]. This disrupts the normal negative feedback loop, where glucocorticoids inhibit the production of inflammatory mediators. Instead, the immune system develops a resistance to glucocorticoids, resulting in a paradoxical co‐existence of high levels of inflammatory cytokines and glucocorticoids. This dysregulation of the HPA axis and immune system can contribute to the development of depression in cancer patients [130]. In summary, the neuroendocrine system, particularly the HPA axis, plays a crucial role in the bidirectional relationship between inflammation and depression in cancer patients [128, 129]. As such, chronic activation of the HPA axis due to immune dysregulation is a key mechanism underlying the increased incidence of depression in this population. Further, the relationship between immune dysregulation, major depression, and cancer has been the subject of extensive research [131, 132, 133]. Evidence suggests that depression is associated with immune dysregulation, including changes in leucocyte trafficking, lymphocyte function, and markers of immune activation [131, 132]. Major depression has been linked to a threefold higher rate of depression in cancer patients, influencing cancer risk and survival [131, 133, 134]. A study examined the association between depressive symptoms and systemic inflammation biomarkers in patients with advanced non‐small cell lung cancer (NSCLC). The researchers found that higher neutrophil‐to‐lymphocyte ratio (NLR) and platelet‐to‐lymphocyte ratio (PLR), as well as lower advanced lung cancer inflammation index (ALI), were predictive of worse overall survival in NSCLC patients. Importantly, the study also showed that patients with moderate to severe depressive symptoms were 2 to 3 times more likely to have these prognostically poor biomarker levels, even after adjusting for other factors. These findings suggest that depression may contribute to the inflammatory dysregulation already present in advanced NSCLC, potentially impacting treatment responses and survival [134]. Further research is needed to understand the biobehavioral mechanisms by which depression may influence disease progression in NSCLC.

Additionally, dysregulation of inflammatory and immune pathways in both depression and cancer has been observed, with evidence of increased expression of proinflammatory cytokines and reductions in immune cell activity [131]. A review by Barreto et al. illustrated and provided evidence of the potential role of tryptophan catabolites (TRYCATs) along the indoleamine 2,3‐dioxygenase (IDO) pathway as a biological link between depression and cancer. Both depression and cancer are associated with dysregulation of inflammatory and immune pathways. IDO, the rate‐limiting enzyme of the TRYCAT pathway, is induced by pro‐inflammatory cytokines and catabolizes tryptophan, producing neuroactive and immune‐modulating compounds. Increased IDO activity in tumor microenvironments is linked to tumor cell escape from immune surveillance. and could be a potential pharmacological target for treating comorbid depression and cancer [135]. Furthermore, the gut microbiome has been implicated in the relationship between cancer symptoms and immune function, specifically in relation to “psychoneurological” symptoms such as depression [133, 136, 137]. Overall, all these researches suggests complex and bidirectional relationships between immune dysregulation, depression, and cancer, emphasizing the need for further investigation into the underlying mechanisms and potential interventions [131, 132, 133].

#### 2.3.3 Genetic and Epigenetic Influences

##### 2.3.3.1 Genetic Markers Associated With Depression in Cancer

Genetic factors play a significant role in the susceptibility to depression among cancer patients. Genome‐wide association studies (GWAS) have identified several genetic variants associated with an increased risk of depression [11].

Several genetic polymorphisms, such as the serotonin transporter‐related promoter region (5‐HTTLPR polymorphism), have been linked to a higher vulnerability for mental disorders and personality traits such as neuroticism in cancer patients [138, 139, 140]. Additionally, the dysbindin gene (DTNBP1) has shown significant association with antidepressant response in patients with major depressive disorder [140, 141]. Research also suggests that mitochondrial DNA and copy number alterations in certain genes, such as EGFR and TYMS, may be associated with depressive symptoms and treatment failure in certain cancers [142, 143]. The identification of genetic markers associated with depression in cancer holds promise for personalized psychiatric interventions and improved patient outcomes [138, 139].

##### 2.3.3.2 Epigenetic Modifications and Their Impact on Depression

Epigenetic modifications, such as DNA methylation and histone acetylation, can regulate gene expression patterns implicated in depression pathogenesis among cancer patients. Stress‐related epigenetic changes without altering the DNA sequence (including DNA methylation, histone modification, chromatin reprogramming, and non‐coding RNA change) may alter the expression of genes involved in neurotransmitter metabolism, stress response, and inflammatory pathways, contributing to the development of depression [144, 145, 146].

Research has shown a strong association between epigenetic modifications and depression in cancer patients [147, 148, 149, 150, 151]. Epigenetic aberrations, including DNA methylation and histone modifications, are linked to the pathogenesis of depression and cancer [149, 150, 151, 152, 153, 154]. Chronic stress and inflammation induce DNA methylation and histone modifications in brain regions, contributing to neurodegenerative disorders and compromised neuroendocrine‐immune‐metabolic adaptive systems [150, 155, 156]. The brain‐derived neurotrophic factor (BDNF) gene, influenced by DNA methylation and genetic profiles, has been independently linked to suicidal ideation in patients with breast cancer [140]. Additionally, environmental factors such as prenatal depression or anxiety, malnutrition, smoking exposure, and psychological stress induce epigenetic changes, which can lead to adverse health effects like depression [150, 156, 157, 158].

A study conducted by Pu et al. explored the link between chronic stress, epigenetic modifications of Hypocretin (HCRT), and depression in cancer progression. Rats exposed to chronic stress exhibited depressive‐like behaviors and had higher tumor loads. HCRT expression was found to be downregulated and its promoter hyper‐methylated in depressed rats. These findings suggest that chronic stress can promote tumorigenesis and cancer progression through epigenetic mechanisms involving HCRT downregulation [159]. Exposure to stress hormones can induce epigenetic modifications that alter the expression of oncogenes and tumor suppressor genes. Research has found that a particular microRNA (miRNA‐145) helps cervical cancer cells resist chemotherapy treatment. Stress hormones like cortisol can reduce the amount of miRNA‐145 in cervical cancer cells infected with HPV [160]. In women with ductal carcinoma in situ, elevated stress levels were correlated with lower histone acetylation in lymphocytes, potentially contributing to a greater risk of tumor spread [161]. Chronic stress was reported to trigger an upregulation of lysine‐specific demethylase 5 (KDM5A), a protein involved in modifying DNA in low‐oxygen environments, thereby promoting tumor progression [162]. While significant advancements have been made in cancer treatment, the development of drug resistance remains a major obstacle. Research indicates that chronic stress may contribute to this resistance by passing down epigenetic changes through generations of cells [163]. Understanding the interplay between epigenetic modifications and depression in cancer may provide insights into potential therapeutic strategies for addressing the mental health challenges faced by cancer patients [149, 151, 155].

## 3 Interplay Between Depression and Cancer

### 3.1 Bidirectional Relationship

Variations in depression or chronic stress prevalence are apparent among different cancer types, with higher rates observed in oropharyngeal, pancreatic, breast, and lung cancers, while colon, gynecological cancers, and lymphoma show relatively lower rates [99].

Chronic stress affects the brain and body's neuroendocrine systems, causing changes in synaptic plasticity, dendritic structure, and the activity of the HPA axis [107, 109, 164]. Through continuous catecholamine release, monocytes from the spleen and bone marrow are drawn to the brain by chronic stress, where they migrate and take on a hyperinflammatory condition [124, 165, 166]. Chronic stress and cancer lead to the activation of specific brain regions by microglia, altering the cerebral microenvironment and reinforcing the neurocircuitry associated with stress [167, 168, 169, 170]. Such physiological alterations may play a role in the onset and progression of cancer by inducing DNA damage, inhibiting tumor suppressor proteins, and encouraging the proliferation of cancer cells [171, 172, 173].

**FIGURE 1:**
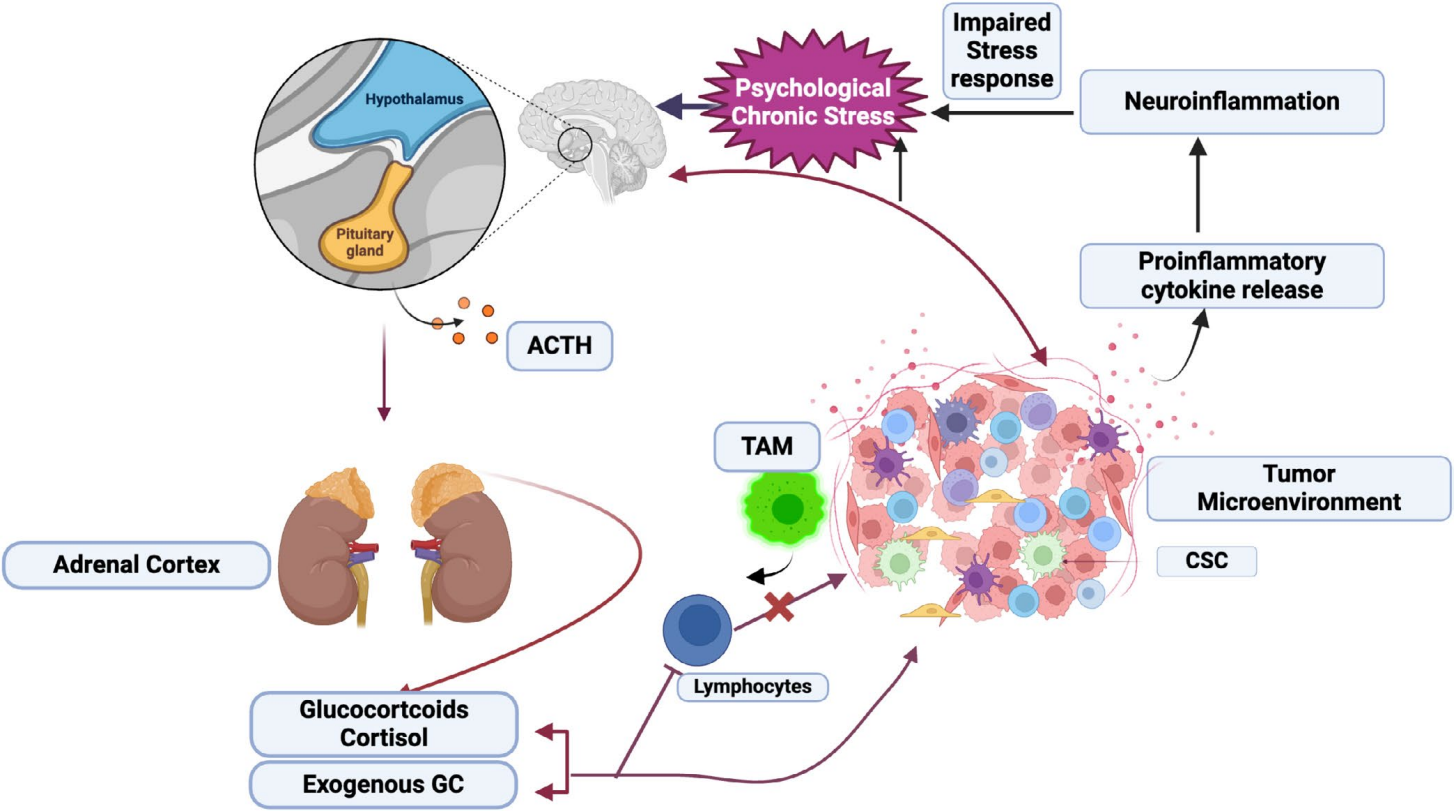
The reciprocal association between cancer and prolonged stress: Hypothalamus‐pituitary–adrenal (HPA) axis is chronically activated in response to chronic stress. Stress hormones can stimulate CSCs by promoting carcinogenesis, supporting the growth and/or progression of cancer, and regulating the microenvironment surrounding tumors through the primary secretion of glucocorticoids (GCs). Moreover, proinflammatory cytokines are produced by both tumors and chronic stress, and these can lead to neuroinflammation and change how the body reacts to stress. Another factor that could lead to a persistent proinflammatory state is anti‐cancer medication [175, 180, 181, 182] (Created in BioRender: https:/biorender.com/h19h122).

Additionally, chronic stress modifies the tumor microenvironment (TME), stimulating angiogenesis, tumor growth, and malignancy. It also impacts immune cell dynamics within the tumor vicinity, disrupting hematopoietic balance and impairing both innate and adaptive immune defenses, thereby facilitating cancer progression [124, 174, 175]. Within the stress signaling network, endoplasmic reticulum (ER) stress emerges as a crucial regulator of CSCs [176]. Chronic psychological stress triggers ER stress, potentially disrupting the connection between cancer stemness and stress [26, 177, 178, 179]. Specifically, chronic stress was found to stimulate lactate production in breast cancer by elevating lactate dehydrogenase A (LDHA). This increased lactate production lowered pH levels, which stabilizes the Myc protein, thereby promoting stem‐like properties in breast cancer cells. One of the symptoms of long‐term psychological stress is elevated plasma glucocorticoid levels [26]. According to related research, cancer patients' increased levels of glucocorticoids may mediate the release of inflammatory factors and tumor immunosurveillance, which in turn may promote tumor heterogeneity and metastasis. Notably, research conducted in the previous 2 years has revealed that glucocorticoids (GCs) and glucocorticoid receptors (GRs) are critical for the regulation of CSCs as shown in Figure 1. By lowering the expression of YAP protein, GR antagonism prevented the production of breast CSCs [180, 181]. Furthermore, GCs facilitated the maintenance of breast CSCs, cell survival, metastasis, and chemotherapy resistance by activating the interaction between GR and domain transcription factor 4 (TEAD4) [183]. Therefore, the CSCs signaling network may have its origin in the elevated glucocorticoids brought on by long‐term psychological disorders. This connection underscores the importance of understanding the biological mechanisms linking stress, inflammation, and cancer progression to develop effective interventions.

### 3.2 Impact on Treatment Adherence and Prognosis

One common pathophysiological process that underlies several chronic illnesses, such as cancer and stress, is persistent low‐grade inflammation [175]. Persistent psychological stress leads to a noticeable and long‐lasting rise in pro‐inflammatory factors in the bloodstream, resulting in low‐grade inflammation in the brain and peripheral tissues [184, 185]. Chronic inflammation and cancer are intricately linked, driven by common signaling pathways such as NF‐kB, STAT3, and mTOR, which regulate proinflammatory cytokine production, creating a self‐sustaining feedback loop [186]. The activation of NF‐kB and STAT3 in concert results in the upregulation of the FAT10 gene, which in turn inhibits the function of the tumor suppressor p53 [187]. The majority of human malignancies involve the TP53 gene mutation, which usually results in the inactivation of the tumor suppressor p53. Research has demonstrated that the p53 protein mutant promotes the survival of cancer cells through increased intracellular reactive oxygen species (ROS) production, proinflammatory cytokine secretion, mTOR signaling activation, decreased autophagic activity, and increased expression of uncoupling protein 2 (UCP2) [188]. Tumor growth, metastasis, and neuroinflammation are further encouraged by proinflammatory cytokine production, which is further exacerbated by stress or hypoxia related to cancer, metabolic alterations, and anticancer therapy [189]. Microglial activation, disruption of the blood–brain barrier, and monocyte trafficking are some of the mechanisms involved in neuroinflammation [166, 190]. As an immuno‐inflammatory condition, depression may play a crucial role in the relationship between inflammatory mediators and cancer, according to studies and reviews [191, 192, 193, 194, 195, 196, 197]. Depression in cancer patients can be classified as an immuno‐inflammatory disorder, associated with elevated levels of proinflammatory mediators such as cytokines and acute phase proteins like CRP and haptoglobin. This association with depression is evident due to immune system activation across various cancer types, triggered by tumor cells releasing cytokines, chemokines, and growth factors, in addition to treatments and psychological stress [193, 198]. Tumor cells release cytokines like IL‐6, CRP, and TNF‐α, as well as chemokines, angiogenic factors, and growth factors, inducing inflammatory responses that can promote or inhibit tumor growth. Treatments like surgery, chemotherapy, and radiotherapy associated with cancer, alongside psychological stress, can trigger the production of proinflammatory cytokines such as IL‐1, INF‐α, IL‐6, and TNF‐α [199, 200, 201]. Consequently, this stimulation could result in elevated levels of inflammatory biomarkers in patients with both cancer and depression compared to those without depression, as evidenced by IL‐6 concentrations [118]. As such, depression among cancer patients is a complex phenomenon influenced by various factors including cancer type, treatment modalities, individual susceptibility, and the interplay between psychological and physiological mechanisms. Recognizing depression as an immuno‐inflammatory disorder sheds light on its intricate etiology and underscores the importance of holistic approaches in managing the mental health aspects of cancer care. Further exploration of the intricate relationships between depression, cancer, and inflammation holds promise for advancing our comprehension and treatment of this prevalent comorbidity.

## 4 Management Strategies for Depression in Cancer Patients

### 4.1 Pharmacological Interventions

#### 4.1.1 Antidepressant Therapy

Antidepressant medications, such as selective serotonin reuptake inhibitors (SSRIs) and serotonin‐norepinephrine reuptake inhibitors (SNRIs), are commonly prescribed to alleviate depressive symptoms in cancer patients [202]. Evidence from majority reviews [202, 203, 204, 205] suggest that antidepressants are more effective than placebo (majority evidence on SSRIs) with one review suggesting no difference between the both groups [206]. Although the overall quality of evidence is poor, some recommendations suggest the use of antidepressants in the treatment of depression, especially in severe cases [202, 204]; while some withhold from making any general recommendations [203, 206]. None suggest a general contra‐indication for antidepressants in cancer patients. There is emerging evidence suggesting the anti‐tumor role of antidepressants [207, 208, 209].

Inflammation contributes to both cancer and psychiatric conditions, with cancer patients facing an increased risk of psychiatric disorders following diagnosis. However, the impact of non‐steroidal anti‐inflammatory drugs (NSAIDs) on subsequent psychiatric disorders remain uncertain. Recent studies have found that compared to non‐users, individuals using aspirin alone had a lower incidence of depression, anxiety, and stress‐related disorders [210]. Further investigations also showed an association between NSAIDs and incident depression among older cancer survivors with osteoarthritis, indicating a nuanced relationship between cumulative NSAID days and incident depression [211].

#### 4.1.2 Adjunctive Medications for Symptom Management

In addition to antidepressants, adjunctive medications have been described in the literature to manage specific symptoms associated with depression in cancer patients. For example, benzodiazepines or buspirone for anxiety symptoms; and trazodone or mirtazapine for sleep disturbances [212].

### 4.2 Role of Psychological Intervention in Cancer Patients

Several psychological interventions have demonstrated efficacy, including relaxation techniques, cognitive‐behavioral therapy (CBT), mindfulness‐based stress reduction (MBSR), acceptance and commitment therapy (ACT), telephonic and web‐based interventions, physical exercise, yoga, and other complementary therapeutic approaches [213, 214, 215, 216, 217]. Studies exploring these interventions for cancer patients have demonstrated benefits in improving psychological and physical symptoms (improvements in fatigue, insomnia, depression, anxiety, pain, distress, and overall quality of life), with some also evaluating changes in biomarkers (summarized in Table 2) [249, 250, 251].

**TABLE 2:** Effect of psychological interventions on various biomarkers.

Type of psychological intervention	Effect on biomarkers
Relaxation techniques	Increase in T‐cell proliferation [218] Increase in mature T cells, NK cells, LAK cells, IL‐1𝛃, CD4/CD8 ratio [219] Reduction of activity related to transcription control pathways involved in adrenergic and glucocorticoid signaling, pro‐inflammatory signaling (NFkB), pro‐malignant signaling (ETS1, STAT and GATA families) [220]. Increased CD4+ T cell activity, M1 macrophage polarization and epithelial‐to‐mesenchymal‐transition (EMT) signature [219]
Cognitive Behavioral Stress Management (CBSM) and Cognitive Behavioral Therapy (CBT)	Increased lymphoproliferation^a^ [217, 221] Increase in IFN‐𝛄, NK cells, IL‐4, IL‐10 and IL‐1𝛃 production [221, 222] Higher Th1 cytokine (IL‐2 and IFN‐𝛄) production and IL‐2:IL‐4 ratio [223] Decrease in serum cortisol [224, 225, 226]
Mindfulness‐based stress reduction (MBSR)	Reduction in levels of proinflammatory cytokines (IFN‐𝛄, TNF) with increase in anti‐inflammatory cytokines (IL‐4, IL‐10), with a shift from Th‐1 (proinflammatory) to Th‐2 (anti‐inflammatory) [227] Reduced salivary cortisol and IL‐6 [228] Increase in heart rate (HR) variability^b^ [229, 230] Telomere Length (TL) was maintained (reduction in cancer patients) [216, 231]
Acceptance and Commitment Therapy (ACT)	Reduced hsCRP and IL‐1Ra levels^a^ [232]
Complementary and alternative medicine therapy—Yoga intervention	Decrease in IgA, increase in CD‐56 T cells [233] Decrease in cortisol levels [234]
Exercise therapy	Reduced levels of IL6, TNF‐α, IL‐10, and leptin [235, 236, 237, 238, 239]
Other complementary and alternative medicine therapies (auricular point acupressure, medical Qigong therapy, body mind spirit therapy, individual massage sessions, hand‐based massage, clown intervention, music therapy)	Reduced CRP levels [240] Reduced cortisol levels [241] Increased NK cells [242] Reduction in salivary Chromogranin‐A [243] Greater HR variability^b^ [244, 245] Greater telomerase activity [246] Decrease in soluble IL‐4 receptor [246]
Telephonic and web‐based interventions	Reduction in IL‐10 [247] Reduction in IL‐6, IL‐1𝛃, IL‐1⍺, IL‐8 and increase in NK cells [248]

^a^ Few studies found no change in inflammatory biomarkers and cortisol levels.

^b^ Higher heart rate variability has been reported in healthy individuals.

Relaxation techniques, such as deep breathing and progressive muscle relaxation, have been shown to relieve both psychological and physiological tension, thereby reducing stress and improving depressive symptoms and quality of life (QOL) in cancer patients [213, 252]. Cognitive‐behavioral therapy (CBT) targets maladaptive cognitive patterns and facilitates emotional regulation, with proven efficacy in alleviating depression and enhancing QOL [217, 253]. Mindfulness‐based stress reduction (MBSR), a structured intervention incorporating meditation, yoga, and related practices, has been demonstrated to effectively reduce loneliness, anxiety, and depression in cancer patients [216, 223]. Similarly, acceptance and commitment therapy (ACT) promotes psychological flexibility by encouraging patients to accept distressing thoughts without becoming overwhelmed. ACT has been found to be comparable to CBT in its ability to improve mood and QOL [254]. Furthermore, exercise therapy may offer modest benefits for depression in cancer patients. Although limited research focuses on depression as a primary outcome, meta‐analytic findings suggest that exercise can reduce pain, alleviate fatigue, and improve QOL in cancer survivors [215, 255].

As such, psychological interventions tailored to the unique needs of cancer patients offer significant potential in alleviating psychological distress, improving quality of life, and influencing immune function, as demonstrated by changes in physiological (reduced heart rate and heart rate variability) [229, 230] and immunological biomarkers (changes in lymphoproliferation and cytokine production [IL6, TNF‐α, IL‐10] [228, 235, 236, 237, 238], decreased serum cortisol and leptin levels, increased NK cell activity, and telomere length stabilization) [216, 219, 221, 222, 224, 231, 235].

## 5 Clinical Implications

### 5.1 Screening and Assessment Strategies

Implementing routine screening and assessment protocols for depression in cancer patients is essential for early detection and intervention. Validated tools such as the Patient Health Questionnaire (PHQ), Beck Depression Inventory‐ii (BDI‐II), and Hospital Anxiety and Depression Scale (HADS) can aid in identifying patients at risk of depression. Clinicians should also consider factors such as cancer type, treatment stage, and comorbidities when assessing depression risk [256].

### 5.2 Tailoring Treatment Approaches

Tailoring treatment approaches to the individual needs and preferences of cancer patients is crucial for optimizing outcomes. Clinicians should consider factors such as patient preferences, treatment side effects, and psychosocial support systems when selecting interventions. A personalized approach that integrates pharmacological, psychotherapeutic, and complementary therapies may enhance treatment adherence and effectiveness [257].

### 5.3 Multidisciplinary Care

Collaborative care models involving multidisciplinary teams comprising oncologists, psychiatrists, psychologists, social workers, and other healthcare professionals can facilitate comprehensive management of depression in cancer patients. CaLM Model provides coordinated, multidisciplinary treatment, which includes nutrition, genetic counselling, pharmacy, psychiatric, and financial and fertility navigation [258]. Multidisciplinary care can successfully enhance patients' quality of life by lowering their feelings of depression and anxiety and enabling them to benefit from comprehensive social support [259].

## 6 Future Directions

### 6.1 Emerging Research Areas

Future research should focus on elucidating the underlying mechanisms linking depression and cancer, including neurobiological, immunological, and genetic factors. Exploring novel treatment modalities, such as immune‐based therapies and targeted interventions, may provide new avenues for managing depression in cancer patients. Additionally, investigating the impact of emerging technologies, such as telemedicine and digital health interventions, on depression management holds promise for improving access to care [260].

### 6.2 Challenges and Opportunities

Addressing barriers to depression care in cancer patients, including stigma, lack of awareness, limited access to mental health services, and treatment‐related side effects, remains a challenge [261, 262]. Opportunities exist to integrate depression screening and management into routine oncology practice through education, training, and policy initiatives [263]. Collaborative efforts involving patients, caregivers, advocacy groups, and healthcare organizations are essential for advancing depression care in cancer settings [264, 265].

## 7 Study Limitations

A comprehensive search strategy across multiple research databases was used. However, it is possible that some relevant studies were inadvertently missing, particularly those with negative or null results. This has the potential to distort review results by overstating the correlation between depression and cancer [266]. This review therefore may not have captured the full breadth and variability of the content included within these interventions, especially those targeting the treatment methods and holistic care plans. The biological interplay between cancer and depression is still needed to be fully understood. Factors such as inflammation, neuroendocrine dysregulation, and genetic predisposition may play roles, but research in this area is still evolving.

## 8 Conclusion

Depression is a prevalent and complex comorbidity in cancer patients, with significant implications for clinical practice and patients. Depression in cancer patients is influenced by a complex interplay of neurobiological, immunological, genetic, and epigenetic factors. Dysregulation of neurotransmitter systems, dysfunction of the HPA axis, inflammatory responses, immune system modulation, genetic susceptibility, and epigenetic modifications contribute to the pathophysiology of depression in this population. Chronic stress can lead to the sustained release of proinflammatory cytokines, maintaining cells in an inflammatory state that influences immune responses and inflammation associated with cancer progression. The observed genetic correlation and potential relationship between stress‐induced depression and cancer highlight the complex interplay between chronic stress, inflammation, immune responses, and cancer development. As discussed above, from relaxation techniques and CBT to exercise therapy and digital interventions, a diverse array of approaches has shown promise in addressing the multifaceted challenges faced by cancer patients. By incorporating these interventions into comprehensive cancer care, healthcare providers can better support patients in navigating the emotional and psychological aspects of their illness, ultimately enhancing their overall well‐being and treatment outcomes. As such, the management of depression in cancer patients involves a multidisciplinary approach encompassing pharmacological interventions, psychotherapeutic approaches, and integrative therapies. Antidepressant therapy, CBT, mindfulness‐based interventions, and exercise programs are among the strategies used to alleviate depressive symptoms and improve psychological well‐being in this vulnerable population.

**FIGURE 2:**
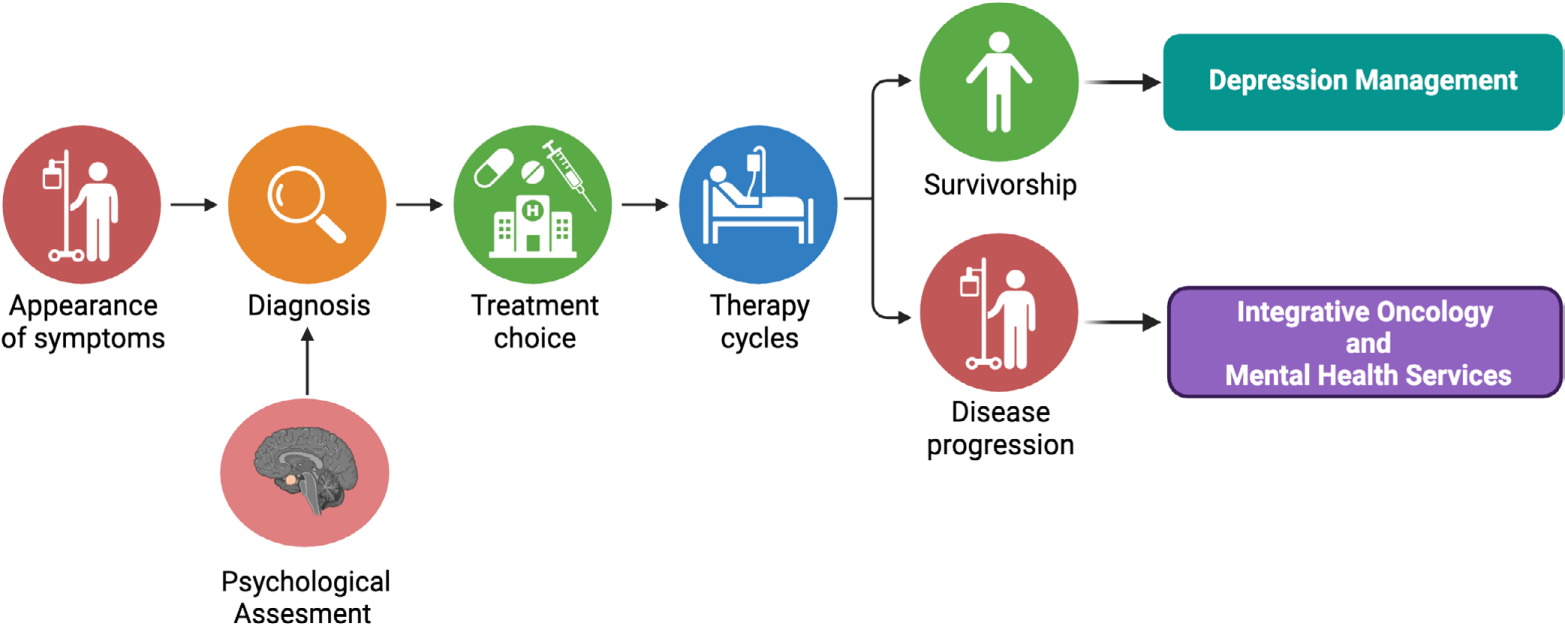
The essential components required to construct a program for coping with cancer are emphasized (The image has been inspired by Cortiana et al. [28]) (Created in BioRender: https:/biorender.com/h19h122).

Further research into the psychobiological underpinnings of depression in cancer patients is warranted to inform the development of targeted interventions aimed at improving mental health outcomes in this vulnerable population. Screening, assessment, and tailored treatment approaches are essential for addressing depression in this population [267]. Multidisciplinary care models that integrate oncology and mental health services can improve depression management and quality of life for cancer patients as summarized in Figure 2 [28, 258]. Researchers should prioritize understanding the mechanisms underlying depression in cancer patients and exploring innovative approaches to prevention and treatment [99]. Clinicians should advocate for the integration of mental health services into cancer care settings and collaborate with interdisciplinary teams to enhance depression screening and management [259]. By addressing the psychosocial needs of cancer patients, healthcare providers can optimize outcomes and improve the overall patient experience.

## Author Contributions

The authors declare that they have made substantial contributions to this manuscript. Joyeeta Talukdar, Megha, Hemant Choudhary and Pratap Sharan were involved in the conceptualization, analysis, and interpretation of data and critical revision of this manuscript. Pratap Sharan, Sushma Bhatnagar, Subhradip Karmakar, Anuja Pandit and Ashwani Kumar Mishra gave final approval for publication and agreed to be responsible for all aspects of the work.

## Ethics Statement

The authors have nothing to report.

## Conflicts of Interest

The authors declare no conflicts of interest.

## Data Availability

Data sharing is not applicable to this article as no datasets were generated or analysed during the current study.

## References

[1] L. D. Godoy , M. T. Rossignoli , P. Delfino‐Pereira , N. Garcia‐Cairasco , and E. H. de Lima Umeoka , “A Comprehensive Overview on Stress Neurobiology: Basic Concepts and Clinical Implications,” Frontiers in Behavioral Neuroscience 12 (2018): 127. 30034327 10.3389/fnbeh.2018.00127 PMC6043787

[2] K. Herskowitz , B. P. Bode , E. R. Block , and W. W. Souba , “The Effects of Endotoxin on Glutamine Transport by Pulmonary Artery Endothelial Cells,” Journal of Surgical Research 50, no. 4 (April 1991): 356–361. 2020187 10.1016/0022-4804(91)90203-x

[3] W. C. Poller , J. Downey , A. A. Mooslechner , et al., “Brain Motor and Fear Circuits Regulate Leukocytes During Acute Stress,” Nature 607, no. 7919 (July 2022): 578–584. 35636458 10.1038/s41586-022-04890-z PMC9798885

[4] F. S. Dhabhar , “Effects of Stress on Immune Function: The Good, the Bad, and the Beautiful,” Immunologic Research 58, no. 2–3 (May 2014): 193–210. 24798553 10.1007/s12026-014-8517-0

[5] P. J. Cowen and M. Browning , “What has Serotonin to Do With Depression?,” World Psychiatry 14, no. 2 (June 2015): 158–160. 26043325 10.1002/wps.20229 PMC4471964

[6] P. L. Delgado , “Depression: The Case for a Monoamine Deficiency,” Journal of Clinical Psychiatry 61, no. Suppl6 (2000): 7–11. 10775018

[7] W. C. Drevets , “Neuroimaging Studies of Mood Disorders,” Biological Psychiatry 48, no. 8 (October 2000): 813–829. 11063977 10.1016/s0006-3223(00)01020-9

[8] A. H. Miller and C. L. Raison , “The Role of Inflammation in Depression: From Evolutionary Imperative to Modern Treatment Target,” Nature Reviews. Immunology 16, no. 1 (January 2016): 22–34. 10.1038/nri.2015.5 PMC5542678 26711676

[9] C. M. Pariante and S. L. Lightman , “The HPA Axis in Major Depression: Classical Theories and New Developments,” Trends in Neurosciences 31, no. 9 (September 2008): 464–468. 18675469 10.1016/j.tins.2008.06.006

[10] R. M. Sapolsky , L. M. Romero , and A. U. Munck , “How Do Glucocorticoids Influence Stress Responses? Integrating Permissive, Suppressive, Stimulatory, and Preparative Actions,” Endocrine Reviews 21, no. 1 (February 2000): 55–89. 10696570 10.1210/edrv.21.1.0389

[11] N. R. Wray , S. Ripke , M. Mattheisen , et al., “Genome‐Wide Association Analyses Identify 44 Risk Variants and Refine the Genetic Architecture of Major Depression,” Nature Genetics 50, no. 5 (May 2018): 668–681. 29700475 10.1038/s41588-018-0090-3 PMC5934326

[12] M. Demers , G. L. Suidan , N. Andrews , K. Martinod , J. E. Cabral , and D. D. Wagner , “Solid Peripheral Tumor Leads to Systemic Inflammation, Astrocyte Activation and Signs of Behavioral Despair in Mice,” PLoS One 13, no. 11 (2018): e0207241. 30439993 10.1371/journal.pone.0207241 PMC6237350

[13] W. E. Nolten , D. Goldstein , M. Lindstrom , et al., “Effects of Cytokines on the Pituitary‐Adrenal Axis in Cancer Patients,” Journal of Interferon Research 13, no. 5 (October 1993): 349–357. 8301155 10.1089/jir.1993.13.349

[14] A. Pitman , S. Suleman , N. Hyde , and A. Hodgkiss , “Depression and Anxiety in Patients With Cancer,” BMJ 25, no. 361 (April 2018): k1415. 10.1136/bmj.k1415 29695476

[15] J. Walker , C. Holm Hansen , P. Martin , et al., “Prevalence of Depression in Adults With Cancer: A Systematic Review,” Annals of Oncology 24, no. 4 (April 2013): 895–900. 23175625 10.1093/annonc/mds575

[16] J. Zhu , F. Fang , A. Sjölander , K. Fall , H. O. Adami , and U. Valdimarsdóttir , “First‐Onset Mental Disorders After Cancer Diagnosis and Cancer‐Specific Mortality: A Nationwide Cohort Study,” Annals of Oncology 28, no. 8 (August 2017): 1964–1969. 28525559 10.1093/annonc/mdx265

[17] Z. Klaassen , C. J. D. Wallis , H. Goldberg , et al., “The Impact of Psychiatric Utilisation Prior to Cancer Diagnosis on Survival of Solid Organ Malignancies,” British Journal of Cancer 120, no. 8 (2019): 840–847. 30837680 10.1038/s41416-019-0390-0 PMC6474265

[18] D. Spiegel and J. Giese‐Davis , “Depression and Cancer: Mechanisms and Disease Progression,” Biological Psychiatry 54, no. 3 (August 2003): 269–282, https://www.sciencedirect.com/science/article/pii/S0006322303005663. 12893103 10.1016/s0006-3223(03)00566-3

[19] D. J. Newport and C. B. Nemeroff , “Assessment and Treatment of Depression in the Cancer Patient,” Journal of Psychosomatic Research 45, no. 3 (September 1998): 215–237. 9776368 10.1016/s0022-3999(98)00011-7

[20] J. S. McDaniel , D. L. Musselman , M. R. Porter , D. A. Reed , and C. B. Nemeroff , “Depression in Patients With Cancer. Diagnosis, Biology, and Treatment,” Archives of General Psychiatry 52, no. 2 (February 1995): 89–99. 7848055 10.1001/archpsyc.1995.03950140007002

[21] B. Burkholder , R. Y. Huang , R. Burgess , et al., “Tumor‐Induced Perturbations of Cytokines and Immune Cell Networks,” Biochimica et Biophysica Acta 1845, no. 2 (April 2014): 182–201. 24440852 10.1016/j.bbcan.2014.01.004

[22] A. Z. Ayob and T. S. Ramasamy , “Cancer Stem Cells as Key Drivers of Tumour Progression,” Journal of Biomedical Science 25, no. 1 (March 2018): 20, 10.1186/s12929-018-0426-4. 29506506 PMC5838954

[23] Q. Liu , Z. Guo , G. Li , et al., “Cancer Stem Cells and Their Niche in Cancer Progression and Therapy,” Cancer Cell International 23 (December 2023): 305, 10.1186/s12935-023-03130-2. 38041196 PMC10693166

[24] X. Chu , W. Tian , J. Ning , et al., “Cancer Stem Cells: Advances in Knowledge and Implications for Cancer Therapy,” 9, no. 1 (July 2024), 1–63, https://www.nature.com/articles/s41392‐024‐01851‐y. 10.1038/s41392-024-01851-y PMC11224386 38965243

[25] J. Yan , Y. Chen , M. Luo , et al., “Chronic Stress in Solid Tumor Development: From Mechanisms to Interventions,” Journal of Biomedical Science 30, no. 1 (2023): 1–25, 10.1186/s12929-023-00903-9. 36707854 PMC9883141

[26] B. Cui , Y. Luo , P. Tian , et al., “Stress‐Induced Epinephrine Enhances Lactate Dehydrogenase A and Promotes Breast Cancer Stem‐Like Cells,” Journal of Clinical Investigation 129, no. 3 (March 2019): 1030–1046. 30688660 10.1172/JCI121685 PMC6391112

[27] L. Yang , P. Shi , G. Zhao , et al., “Targeting Cancer Stem Cell Pathways for Cancer Therapy,” Signal Transduction and Targeted Therapy 5, no. 1 (February 2020): 1–35, https://www.nature.com/articles/s41392‐020‐0110‐5. 32296030 10.1038/s41392-020-0110-5 PMC7005297

[28] V. Cortiana , R. H. Abbas , S. Nadar , et al., “Reviewing the Landscape of Cancer Survivorship: Insights From Dr. Lidia Schapira's Programs and Beyond,” Cancers (Basel) 16, no. 6 (March 2024): 1216. 38539549 10.3390/cancers16061216 PMC10969740

[29] C. Sandi and J. Haller , “Stress and the Social Brain: Behavioural Effects and Neurobiological Mechanisms,” Nature Reviews. Neuroscience 16, no. 5 (May 2015): 290–304. 25891510 10.1038/nrn3918

[30] C. Alonso , M. Guilarte , M. Vicario , et al., “Maladaptive Intestinal Epithelial Responses to Life Stress May Predispose Healthy Women to Gut Mucosal Inflammation,” Gastroenterology 135, no. 1 (July 2008): 163–172.e1. 18455999 10.1053/j.gastro.2008.03.036

[31] J. Santos and M. H. Perdue , “Stress and Neuroimmune Regulation of Gut Mucosal Function,” Gut 47, no. Suppl 4 (December 2000): iv49–iv51, discussion iv52. 11076912 10.1136/gut.47.suppl_4.iv49 PMC1766810

[32] Q. G. Zhou , L. J. Zhu , C. Chen , et al., “Hippocampal Neuronal Nitric Oxide Synthase Mediates the Stress‐Related Depressive Behaviors of Glucocorticoids by Downregulating Glucocorticoid Receptor,” Journal of Neuroscience 31, no. 21 (May 2011): 7579–7590. 21613472 10.1523/JNEUROSCI.0004-11.2011 PMC6633122

[33] C. Menard , M. L. Pfau , G. E. Hodes , et al., “Social Stress Induces Neurovascular Pathology Promoting Depression,” Nature Neuroscience 20, no. 12 (December 2017): 1752–1760. 29184215 10.1038/s41593-017-0010-3 PMC5726568

[34] K. Cooper , F. Campbell , S. Harnan , and A. Sutton , “Association Between Stress, Depression or Anxiety and Cancer: Rapid Review of Reviews,” Comprehensive Psychoneuroendocrinology 16 (November 2023): 100215, https://www.sciencedirect.com/science/article/pii/S2666497623000498. 38108025 10.1016/j.cpnec.2023.100215 PMC10724821

[35] P. B. McEwen , M. Palmer , and D. R. Anderson , “Allostatic Load—A Review of the Literature,” 2019.

[36] M. M. Fidler , S. Gupta , I. Soerjomataram , J. Ferlay , E. Steliarova‐Foucher , and F. Bray , “Cancer Incidence and Mortality Among Young Adults Aged 20‐39 Years Worldwide in 2012: A Population‐Based Study,” Lancet Oncology 18, no. 12 (2017): 1579–1589. 29111259 10.1016/S1470-2045(17)30677-0

[37] C. E. DeSantis , K. D. Miller , A. Goding Sauer , A. Jemal , and R. L. Siegel , “Cancer Statistics for African Americans, 2019,” CA: A Cancer Journal for Clinicians 69, no. 3 (May 2019): 211–233. 30762872 10.3322/caac.21555

[38] A. Mathew , A. Z. Doorenbos , H. Li , M. K. Jang , C. G. Park , and U. G. Bronas , “Allostatic Load in Cancer: A Systematic Review and Mini Meta‐Analysis,” Biological Research for Nursing 23, no. 3 (July 2021): 341–361, https://www.ncbi.nlm.nih.gov/pmc/articles/PMC8755951/. 33138637 10.1177/1099800420969898 PMC8755951

[39] J. Shen , B. F. Fuemmeler , Y. Guan , and H. Zhao , “Association of Allostatic Load and All Cancer Risk in the SWAN Cohort,” Cancers (Basel) 14, no. 13 (June 2022): 3044, https://www.ncbi.nlm.nih.gov/pmc/articles/PMC9264860/. 35804816 10.3390/cancers14133044 PMC9264860

[40] C. L. Niedzwiedz , L. Knifton , K. A. Robb , S. V. Katikireddi , and D. J. Smith , “Depression and Anxiety Among People Living With and Beyond Cancer: A Growing Clinical and Research Priority,” BMC Cancer 19, no. 1 (October 2019): 943. 31604468 10.1186/s12885-019-6181-4 PMC6788022

[41] A. Hurria , C. Rosen , C. Hudis , et al., “Cognitive Function of Older Patients Receiving Adjuvant Chemotherapy for Breast Cancer: A Pilot Prospective Longitudinal Study,” Journal of the American Geriatrics Society 54, no. 6 (June 2006): 925–931. 16776787 10.1111/j.1532-5415.2006.00732.x

[42] F. Mols and J. Denollet , “Type D Personality Among Noncardiovascular Patient Populations: A Systematic Review,” General Hospital Psychiatry 32, no. 1 (2010): 66–72. 20114130 10.1016/j.genhosppsych.2009.09.010

[43] M. Piccinelli and G. Wilkinson , “Gender Differences in Depression: Critical Review,” British Journal of Psychiatry 177, no. 6 (December 2000): 486–492, https://www.cambridge.org/core/journals/the‐british‐journal‐of‐psychiatry/article/gender‐differences‐in‐depression/0770B51752F17A5A081F9878B0952608. 10.1192/bjp.177.6.486 11102321

[44] D. P. Stark and A. House , “Anxiety in Cancer Patients,” British Journal of Cancer 83, no. 10 (November 2000): 1261–1267. 11044347 10.1054/bjoc.2000.1405 PMC2408796

[45] S. M. Strayhorn‐Carter , K. Batai , and F. C. Gachupin , “Types of Racism and Health Disparities and Inequalities Among Cancer Patients: An Editorial Reflection of Articles in This Special Issue of IJERPH,” International Journal of Environmental Research and Public Health 21, no. 6 (June 2024): 785, https://www.mdpi.com/1660‐4601/21/6/785. 38929031 10.3390/ijerph21060785 PMC11203658

[46] C. Boettger , “Cancer Patients' Spiritual Well‐Being, Religious Coping, and Comfort in Communicating With Physicians,” Walden Dissertations and Doctoral Studies, 2024, https://scholarworks.waldenu.edu/dissertations/15520.

[47] J. M. Ussher , K. Allison , J. Perz , and R. Power , “The Out With Cancer Study Team. LGBTQI Cancer Patients' Quality of Life and Distress: A Comparison by Gender, Sexuality, Age, Cancer Type and Geographical Remoteness,” Frontiers in Oncology 12 (September 2022): 873642, 10.3389/fonc.2022.873642/full. 36203463 PMC9530284

[48] M. Matzka , H. Mayer , S. Köck‐Hódi , et al., “Relationship Between Resilience, Psychological Distress and Physical Activity in Cancer Patients: A Cross‐Sectional Observation Study,” PLoS One 11, no. 4 (2016): e0154496, 10.1371/journal.pone.0154496. 27124466 PMC4849643

[49] C. Wang , X. Qiu , X. Yang , J. Mao , and Q. Li , “Factors Influencing Social Isolation Among Cancer Patients: A Systematic Review,” Healthcare 12, no. 10 (January 2024): 1042, https://www.mdpi.com/2227‐9032/12/10/1042. 38786452 10.3390/healthcare12101042 PMC11120751

[50] P. W. Gold , “The Organization of the Stress System and Its Dysregulation in Depressive Illness,” Molecular Psychiatry 20, no. 1 (February 2015): 32–47. 25486982 10.1038/mp.2014.163

[51] J. W. Buckholtz and A. Meyer‐Lindenberg , “MAOA and the Neurogenetic Architecture of Human Aggression,” Trends in Neurosciences 31, no. 3 (March 2008): 120–129. 18258310 10.1016/j.tins.2007.12.006

[52] A. Caspi , K. Sugden , T. E. Moffitt , et al., “Influence of Life Stress on Depression: Moderation by a Polymorphism in the 5‐HTT Gene,” Science 301, no. 5631 (July 2003): 386–389. 12869766 10.1126/science.1083968

[53] V. Krishnan and E. J. Nestler , “The Molecular Neurobiology of Depression,” Nature 455, no. 7215 (October 2008): 894–902. 18923511 10.1038/nature07455 PMC2721780

[54] R. Zhao , H. Liu , and J. Gao , “Side Effects of Endocrine Therapy Are Associated With Depression and Anxiety in Breast Cancer Patients Accepting Endocrine Therapy: A Cross‐Sectional Study in China,” Frontiers in Psychology 13 (May 2022): 905459, https://www.ncbi.nlm.nih.gov/pmc/articles/PMC9125212/. 35615194 10.3389/fpsyg.2022.905459 PMC9125212

[55] R. Dantzer and K. W. Kelley , “Twenty Years of Research on Cytokine‐Induced Sickness Behavior,” Brain, Behavior, and Immunity 21, no. 2 (February 2007): 153–160. 17088043 10.1016/j.bbi.2006.09.006 PMC1850954

[56] K. L. Syrjala , M. P. Jensen , M. E. Mendoza , J. C. Yi , H. M. Fisher , and F. J. Keefe , “Psychological and Behavioral Approaches to Cancer Pain Management,” Journal of Clinical Oncology 32, no. 16 (June 2014): 1703–1711. 24799497 10.1200/JCO.2013.54.4825 PMC4031190

[57] H. Oers and L. Schlebusch , “Indicators of Psychological Distress and Body Image Disorders in Female Patients With Breast Cancer,” Journal of Mind and Medical Sciences 7, no. 2 (September 2020): 179–187, https://scholar.valpo.edu/jmms/vol7/iss2/9.

[58] K. Mystakidou , E. Tsilika , E. Parpa , et al., “Illness‐Related Hopelessness in Advanced Cancer: Influence of Anxiety, Depression, and Preparatory Grief,” Archives of Psychiatric Nursing 23, no. 2 (April 2009): 138–147, https://www.sciencedirect.com/science/article/pii/S0883941708000927. 19327556 10.1016/j.apnu.2008.04.008

[59] D. Ikhile , E. Ford , D. Glass , G. Gremesty , and H. van Marwijk , “A Systematic Review of Risk Factors Associated With Depression and Anxiety in Cancer Patients,” PLoS One 19, no. 3 (March 2024): e0296892, 10.1371/journal.pone.0296892. 38551956 PMC10980245

[60] M. Li , “Associations of Body Image With Depressive Symptoms and PTG Among Breast Cancer Patients: The Mediating Role of Social Support,” Frontiers in Psychology 13 (October 2022): 953306, 10.3389/fpsyg.2022.953306/full. 36312105 PMC9614141

[61] E. Kacel , X. Gao , and H. G. Prigerson , “Understanding Bereavement: What Every Oncology Practitioner Should Know,” Journal of Supportive Oncology 9, no. 5 (2011): 172–180, https://www.ncbi.nlm.nih.gov/pmc/articles/PMC3202698/. 22024306 10.1016/j.suponc.2011.04.007 PMC3202698

[62] R. D. Nipp , A. El‐Jawahri , J. N. Fishbein , et al., “The Relationship Between Coping Strategies, Quality of Life, and Mood in Patients With Incurable Cancer,” Cancer 122, no. 13 (July 2016): 2110–2116, https://www.ncbi.nlm.nih.gov/pmc/articles/PMC5160928/. 27089045 10.1002/cncr.30025 PMC5160928

[63] J. W. F. Aarts , L. Deckx , D. L. van Abbema , V. C. G. Tjan‐Heijnen , M. van den Akker , and F. Buntinx , “The Relation Between Depression, Coping and Health Locus of Control: Differences Between Older and Younger Patients, With and Without Cancer,” Psycho‐Oncology 24, no. 8 (August 2015): 950–957. 25644618 10.1002/pon.3748

[64] H. L. Lai , C. M. Hung , C. I. Chen , M. L. Shih , and C. Y. Huang , “Resilience and Coping Styles as Predictors of Health Outcomes in Breast Cancer Patients: A Structural Equation Modelling Analysis,” European Journal of Cancer Care 29, no. 1 (January 2020): e13161. 31475417 10.1111/ecc.13161

[65] S. Grogan , “Body Image and Health: Contemporary Perspectives,” Journal of Health Psychology 11, no. 4 (July 2006): 523–530. 16769732 10.1177/1359105306065013

[66] R. Dev , M. Agosta , B. Fellman , et al., “Coping Strategies and Associated Symptom Burden Among Patients With Advanced Cancer,” Oncologist 29, no. 2 (September 2023): 166–175, https://www.ncbi.nlm.nih.gov/pmc/articles/PMC10836315/. 10.1093/oncolo/oyad253 PMC10836315 37669020

[67] E. C. Fradelos , I. V. Papathanasiou , A. Veneti , et al., “Psychological Distress and Resilience in Women Diagnosed With Breast Cancer in Greece,” Asian Pacific Journal of Cancer Prevention 18, no. 9 (2017): 2545–2550, https://www.ncbi.nlm.nih.gov/pmc/articles/PMC5720664. 28952298 10.22034/APJCP.2017.18.9.2545 PMC5720664

[68] K. K. Manier , L. S. Rowe , J. Welsh , and T. S. Armstrong , “The Impact and Incidence of Altered Body Image in Patients With Head and Neck Tumors: A Systematic Review,” Neuro‐Oncology Practice 5, no. 4 (November 2018): 204–213, https://www.ncbi.nlm.nih.gov/pmc/articles/PMC6656294/. 31386002 10.1093/nop/npy018 PMC6656294

[69] H. J. Eysenck , “Cancer, Personality and Stress: Prediction and Prevention,” Advances in Behaviour Research and Therapy 16, no. 3 (January 1994): 167–215, https://www.sciencedirect.com/science/article/pii/0146640294000018.

[70] L. Grassi , R. Caruso , M. B. Murri , et al., “Association Between Type‐D Personality and Affective (Anxiety, Depression, Post‐Traumatic Stress) Symptoms and Maladaptive Coping in Breast Cancer Patients: A Longitudinal Study,” Clinical Practice and Epidemiology in Mental Health 17, no. Supp‐1 (December 2021): 271–279, https://www.ncbi.nlm.nih.gov/pmc/articles/PMC8985468/. 35444709 10.2174/1745017902117010271 PMC8985468

[71] A. A. Dahl , S. K. Smedsland , K. F. Vandraas , et al., “High Neuroticism Is Associated With Common Late Adverse Effects in a Nationwide Sample of Long‐Term Breast Cancer Survivors,” Breast Cancer Research and Treatment 202, no. 1 (2023): 97–104, https://www.ncbi.nlm.nih.gov/pmc/articles/PMC10504095/. 37528264 10.1007/s10549-023-07055-2 PMC10504095

[72] D. W. Kissane , D. M. Clarke , and A. F. Street , “Demoralization Syndrome—A Relevant Psychiatric Diagnosis for Palliative Care,” Journal of Palliative Care 17, no. 1 (2001): 12–21. 11324179

[73] K. E. Henson , R. Brock , J. Charnock , B. Wickramasinghe , O. Will , and A. Pitman , “Risk of Suicide After Cancer Diagnosis in England,” JAMA Psychiatry 76, no. 1 (January 2019): 51–60. 30476945 10.1001/jamapsychiatry.2018.3181 PMC6583458

[74] S.‐M. Wang , J.‐C. Chang , S.‐C. Weng , M.‐K. Yeh , and C.‐S. Lee , “Risk of Suicide Within 1 Year of Cancer Diagnosis,” International Journal of Cancer 142 (2018): 1986–1993, 10.1002/ijc.31224. 29250783

[75] K. Hodgkinson , P. Butow , G. E. Hunt , et al., “The Development and Evaluation of a Measure to Assess Cancer Survivors' Unmet Supportive Care Needs: The CaSUN (Cancer Survivors' Unmet Needs Measure),” Psycho‐Oncology 16, no. 9 (2007): 796–804, 10.1002/pon.1137. 17177268

[76] M. J. Cordova , M. B. Riba , and D. Spiegel , “Post‐Traumatic Stress Disorder and Cancer,” Lancet Psychiatry 4, no. 4 (April 2017): 330–338. 28109647 10.1016/S2215-0366(17)30014-7 PMC5676567

[77] S. K. Deenadayalan , K. Balakrishnan , and S. Chidambaram , “Factors Associated With Knowledge of Diagnosis, Prognosis & Distress in Cancer Patients Receiving Palliative Care—A Retrospective Cohort Analysis,” Indian Journal of Medical Research 157, no. 6 (June 2023): 568–576. 37530312 10.4103/ijmr.ijmr_2843_21 PMC10466486

[78] C. E. Holden , S. Wheelwright , A. Harle , and R. Wagland , “The Role of Health Literacy in Cancer Care: A Mixed Studies Systematic Review,” PLoS One 16, no. 11 (November 2021): e0259815, https://www.ncbi.nlm.nih.gov/pmc/articles/PMC8589210/. 34767562 10.1371/journal.pone.0259815 PMC8589210

[79] J. G. Grzywacz and J. Fuqua , “The Social Ecology of Health: Leverage Points and Linkages,” Behavioral Medicine 26, no. 3 (2000): 101–115. 11209591 10.1080/08964280009595758

[80] H. E. Leeper , E. Vera , A. Christ , et al., “Association of Employment Status With Symptom Burden and Health‐Related Quality of Life in People Living With Primary CNS Tumors,” Neurology 100, no. 16 (April 2023): e1723–e1736, https://www.ncbi.nlm.nih.gov/pmc/articles/PMC10115506/. 36754634 10.1212/WNL.0000000000207082 PMC10115506

[81] L. J. Su , S. N. O'Connor , and T. C. Chiang , “Association Between Household Income and Self‐Perceived Health Status and Poor Mental and Physical Health Among Cancer Survivors,” Frontiers in Public Health 9 (December 2021): 752868, https://www.ncbi.nlm.nih.gov/pmc/articles/PMC8688689/. 34950625 10.3389/fpubh.2021.752868 PMC8688689

[82] K. G. Nissen , K. Trevino , T. Lange , and H. G. Prigerson , “Family Relationships and Psychosocial Dysfunction Among Family Caregivers of Patients With Advanced Cancer,” Journal of Pain and Symptom Management 52, no. 6 (December 2016): 841–849.e1, https://www.sciencedirect.com/science/article/pii/S088539241630224X. 27521285 10.1016/j.jpainsymman.2016.07.006 PMC5497710

[83] W. Linden , A. Vodermaier , R. Mackenzie , and D. Greig , “Anxiety and Depression After Cancer Diagnosis: Prevalence Rates by Cancer Type, Gender, and Age,” Journal of Affective Disorders 141, no. 2–3 (December 2012): 343–351. 22727334 10.1016/j.jad.2012.03.025

[84] M. Daher , “Cultural Beliefs and Values in Cancer Patients,” Annals of Oncology 23 (April 2012): iii66–iii69, https://www.annalsofoncology.org/article/S0923‐7534(19)38895‐7/fulltext. 10.1093/annonc/mds091 22628419

[85] I. Chidobem , F. Tian , C. Mgbodile , et al., “Assessing the Relationship Between Socioeconomic Status, Race, and Psychological Distress in Cancer Survivors: A Population Based Study,” Current Oncology 29, no. 4 (April 2022): 2575–2582, https://www.ncbi.nlm.nih.gov/pmc/articles/PMC9025824/. 35448185 10.3390/curroncol29040211 PMC9025824

[86] B. N. Uchino , J. T. Cacioppo , and J. K. Kiecolt‐Glaser , “The Relationship Between Social Support and Physiological Processes: A Review With Emphasis on Underlying Mechanisms and Implications for Health,” Psychological Bulletin 119, no. 3 (May 1996): 488–531. 8668748 10.1037/0033-2909.119.3.488

[87] S. N. Price , A. M. Palmer , L. M. Fucito , et al., “Tobacco Use and Cancer‐Related Symptom Burden: Analysis of the US Population Assessment of Tobacco and Health Study,” Cancer 129, no. 15 (August 2023): 2385–2394, https://www.ncbi.nlm.nih.gov/pmc/articles/PMC10593116/. 37211959 10.1002/cncr.34746 PMC10593116

[88] D. Révész , M. J. L. Bours , J. A. Wegdam , et al., “Associations Between Alcohol Consumption and Anxiety, Depression, and Health‐Related Quality of Life in Colorectal Cancer Survivors,” Journal of Cancer Survivorship 16, no. 5 (2022): 988–997, https://www.ncbi.nlm.nih.gov/pmc/articles/PMC9489554/. 34529261 10.1007/s11764-021-01090-y PMC9489554

[89] A. M. Lasserre , M. P. F. Strippoli , P. Marques‐Vidal , et al., “Dietary Patterns Are Differentially Associated With Atypical and Melancholic Subtypes of Depression,” Nutrients 13, no. 3 (February 2021): 768. 33653007 10.3390/nu13030768 PMC7996872

[90] T. Ljungberg , E. Bondza , and C. Lethin , “Evidence of the Importance of Dietary Habits Regarding Depressive Symptoms and Depression,” International Journal of Environmental Research and Public Health 17, no. 5 (March 2020): 1616. 32131552 10.3390/ijerph17051616 PMC7084175

[91] S. Fulton , L. Décarie‐Spain , X. Fioramonti , B. Guiard , and S. Nakajima , “The Menace of Obesity to Depression and Anxiety Prevalence,” Trends in Endocrinology and Metabolism 33, no. 1 (January 2022): 18–35, https://www.sciencedirect.com/science/article/pii/S1043276021002411. 34750064 10.1016/j.tem.2021.10.005

[92] F. S. Luppino , L. M. de Wit , P. F. Bouvy , et al., “Overweight, Obesity, and Depression: A Systematic Review and Meta‐Analysis of Longitudinal Studies,” Archives of General Psychiatry 67, no. 3 (March 2010): 220–229. 20194822 10.1001/archgenpsychiatry.2010.2

[93] M. Ferioli , G. Zauli , A. M. Martelli , et al., “Impact of Physical Exercise in Cancer Survivors During and After Antineoplastic Treatments,” Oncotarget 9, no. 17 (February 2018): 14005–14034, https://www.ncbi.nlm.nih.gov/pmc/articles/PMC5862633/. 29568412 10.18632/oncotarget.24456 PMC5862633

[94] D. Howell , T. K. Oliver , S. Keller‐Olaman , et al., “Sleep Disturbance in Adults With Cancer: A Systematic Review of Evidence for Best Practices in Assessment and Management for Clinical Practice,” Annals of Oncology 25, no. 4 (April 2014): 791–800. 24287882 10.1093/annonc/mdt506

[95] K. O. Anderson , C. J. Getto , T. R. Mendoza , et al., “Fatigue and Sleep Disturbance in Patients With Cancer, Patients With Clinical Depression, and Community‐Dwelling Adults,” Journal of Pain and Symptom Management 25, no. 4 (April 2003): 307–318. 12691682 10.1016/s0885-3924(02)00682-6

[96] M. Pinquart and P. R. Duberstein , “Depression and Cancer Mortality: A Meta‐Analysis,” Psychological Medicine 40, no. 11 (November 2010): 1797–1810. 20085667 10.1017/S0033291709992285 PMC2935927

[97] J. Walker , C. H. Hansen , P. Martin , et al., “Prevalence, Associations, and Adequacy of Treatment of Major Depression in Patients With Cancer: A Cross‐Sectional Analysis of Routinely Collected Clinical Data,” Lancet Psychiatry 1, no. 5 (October 2014): 343–350, https://www.sciencedirect.com/science/article/pii/S221503661470313X. 26360998 10.1016/S2215-0366(14)70313-X

[98] A. Mehnert , E. Brähler , H. Faller , et al., “Four‐Week Prevalence of Mental Disorders in Patients With Cancer Across Major Tumor Entities,” Journal of Clinical Oncology 32, no. 31 (November 2014): 3540–3546. 25287821 10.1200/JCO.2014.56.0086

[99] M. J. Massie , “Prevalence of Depression in Patients With Cancer,” Journal of the National Cancer Institute. Monographs 32 (2004): 57–71. 10.1093/jncimonographs/lgh014 15263042

[100] J. Hamer , R. McDonald , L. Zhang , et al., “Quality of Life (QOL) and Symptom Burden (SB) in Patients With Breast Cancer,” Supportive Care in Cancer 25, no. 2 (February 2017): 409–419. 27696078 10.1007/s00520-016-3417-6

[101] W. F. Pirl , J. A. Greer , L. Traeger , et al., “Depression and Survival in Metastatic Non‐Small‐Cell Lung Cancer: Effects of Early Palliative Care,” Journal of Clinical Oncology 30, no. 12 (April 2012): 1310–1315. 22430269 10.1200/JCO.2011.38.3166 PMC3341144

[102] J. Zabora , K. BrintzenhofeSzoc , B. Curbow , C. Hooker , and S. Piantadosi , “The Prevalence of Psychological Distress by Cancer Site,” Psycho‐Oncology 10, no. 1 (2001): 19–28. 11180574 10.1002/1099-1611(200101/02)10:1<19::aid-pon501>3.0.co;2-6

[103] A. Y. Naser , A. N. Hameed , N. Mustafa , et al., “Depression and Anxiety in Patients With Cancer: A Cross‐Sectional Study,” Frontiers in Psychology 12 (April 2021): 585534, 10.3389/fpsyg.2021.585534/full. 33935849 PMC8081978

[104] K. V. Reinertsen , M. Cvancarova , J. H. Loge , H. Edvardsen , E. Wist , and S. D. Fosså , “Predictors and Course of Chronic Fatigue in Long‐Term Breast Cancer Survivors,” Journal of Cancer Survivorship 4, no. 4 (December 2010): 405–414. 20862614 10.1007/s11764-010-0145-7 PMC2978315

[105] R. J. Chan , L. G. Gordon , C. J. Tan , et al., “Relationships Between Financial Toxicity and Symptom Burden in Cancer Survivors: A Systematic Review,” Journal of Pain and Symptom Management 57, no. 3 (March 2019): 646–660.e1, https://www.sciencedirect.com/science/article/pii/S0885392418311163. 30550833 10.1016/j.jpainsymman.2018.12.003

[106] H. R. Smith , “Depression in Cancer Patients: Pathogenesis, Implications and Treatment (Review),” Oncology Letters 9, no. 4 (2015): 1509–1514. 25788991 10.3892/ol.2015.2944 PMC4356432

[107] B. S. McEwen , C. Nasca , and J. D. Gray , “Stress Effects on Neuronal Structure: Hippocampus, Amygdala, and Prefrontal Cortex,” Neuropsychopharmacology 41, no. 1 (January 2016): 3–23. 26076834 10.1038/npp.2015.171 PMC4677120

[108] D. Patel , S. Anilkumar , S. Chattarji , and B. Buwalda , “Repeated Social Stress Leads to Contrasting Patterns of Structural Plasticity in the Amygdala and Hippocampus,” Behavioural Brain Research 16, no. 347 (July 2018): 314–324. 10.1016/j.bbr.2018.03.034 29580891

[109] J. P. Herman and J. G. Tasker , “Paraventricular Hypothalamic Mechanisms of Chronic Stress Adaptation,” Frontiers in Endocrinology 7 (2016): 137. 27843437 10.3389/fendo.2016.00137 PMC5086584

[110] S. A. Lowrance , A. Ionadi , E. McKay , X. Douglas , and J. D. Johnson , “Sympathetic Nervous System Contributes to Enhanced Corticosterone Levels Following Chronic Stress,” Psychoneuroendocrinology 68 (June 2016): 163–170. 26974501 10.1016/j.psyneuen.2016.02.027 PMC5656452

[111] M. K. Nowakowska , X. Lei , M. R. Wehner , P. G. Corn , S. H. Giordano , and K. T. Nead , “Association of Second‐Generation Antiandrogens With Depression Among Patients With Prostate Cancer,” JAMA Network Open 4, no. 12 (December 2021): e2140803, 10.1001/jamanetworkopen.2021.40803. 34940861 PMC8703250

[112] P. A. Tsao , R. D. Ross , A. S. B. Bohnert , B. Mukherjee , and C. Mev , “Depression, Anxiety, and Patterns of Mental Health Care Among Men With Prostate Cancer Receiving Androgen Deprivation Therapy,” Oncologist 27 (April 2022): 314–322, 10.1093/oncolo/oyab033. 35298660 PMC8982372

[113] A. Alwhaibi , S. Alsanea , B. Almadi , J. Al‐sabhan , and F. D. Alosaimi , “Androgen Deprivation Therapy and Depression in the Prostate Cancer Patients: Review of Risk and Pharmacological Management,” Aging Male 25, no. 1 (December 2022): 101–124, 10.1080/13685538.2022.2053954. 35343371

[114] A. I. Hoogland , H. S. L. Jim , B. D. Gonzalez , et al., “Systemic Inflammation and Symptomatology in Patients With Prostate Cancer Treated With Androgen Deprivation Therapy: Preliminary Findings,” Cancer 127, no. 9 (May 2021): 1476–1482. 33378113 10.1002/cncr.33397 PMC8084887

[115] C. Xiao , A. H. Miller , J. Felger , D. Mister , T. Liu , and M. A. Torres , “Depressive Symptoms and Inflammation Are Independent Risk Factors of Fatigue in Breast Cancer Survivors,” Psychological Medicine 47, no. 10 (July 2017): 1733–1743. 28193310 10.1017/S0033291717000150

[116] J. E. Bower , P. A. Ganz , M. L. Tao , et al., “Inflammatory Biomarkers and Fatigue During Radiation Therapy for Breast and Prostate Cancer,” Clinical Cancer Research 15, no. 17 (August 2009): 5534–5540, 10.1158/1078-0432.CCR-08-2584. 19706826 PMC2884979

[117] C. Schubert , S. Hong , L. Natarajan , P. J. Mills , and J. E. Dimsdale , “The Association Between Fatigue and Inflammatory Marker Levels in Cancer Patients: A Quantitative Review,” Brain, Behavior, and Immunity 21, no. 4 (May 2007): 413–427, https://www.sciencedirect.com/science/article/pii/S0889159106003576. 17178209 10.1016/j.bbi.2006.11.004

[118] C. F. Jehn , D. Kuehnhardt , A. Bartholomae , et al., “Biomarkers of Depression in Cancer Patients,” Cancer 107, no. 11 (December 2006): 2723–2729. 17036362 10.1002/cncr.22294

[119] U. R. Malik , D. F. Makower , and S. Wadler , “Interferon‐Mediated Fatigue,” Cancer 92, no. 6 Suppl (September 2001): 1664–1668. 11598884 10.1002/1097-0142(20010915)92:6+<1664::aid-cncr1494>3.0.co;2-9

[120] D. L. Musselman , D. H. Lawson , J. F. Gumnick , et al., “Paroxetine for the Prevention of Depression Induced by High‐Dose Interferon Alfa,” New England Journal of Medicine 344 (2024): 961–966, 10.1056/NEJM200103293441303. 11274622

[121] H. Miyaoka , T. Otsubo , K. Kamijima , M. Ishii , M. Onuki , and K. Mitamura , “Depression From Interferon Therapy in Patients With Hepatitis C,” American Journal of Psychiatry 156, no. 7 (July 1999): 1120. 10.1176/ajp.156.7.1120 10401474

[122] H. Li , X. Shi , F. Yang , X. Zhang , and F. Li , “Blood Inflammatory Cytokines as Predictors of Depression in Patients With Glioma,” Frontiers in Psychiatry 13 (June 2022): 930985, https://www.ncbi.nlm.nih.gov/pmc/articles/PMC9218211/. 35757220 10.3389/fpsyt.2022.930985 PMC9218211

[123] H. Y. Ho , V. Chin‐Hung Chen , B. S. Tzang , et al., “Circulating Cytokines as Predictors of Depression in Patients With Breast Cancer,” Journal of Psychiatric Research 136 (April 2021): 306–311, https://www.sciencedirect.com/science/article/pii/S0022395621001047. 33636686 10.1016/j.jpsychires.2021.02.037

[124] T. J. Barrett , E. M. Corr , C. van Solingen , et al., “Chronic Stress Primes Innate Immune Responses in Mice and Humans,” Cell Reports 36, no. 10 (September 2021): 109595. 34496250 10.1016/j.celrep.2021.109595 PMC8493594

[125] J. R. Satin , W. Linden , and M. J. Phillips , “Depression as a Predictor of Disease Progression and Mortality in Cancer Patients,” Cancer 115, no. 22 (2009): 5349–5361, 10.1002/cncr.24561. 19753617

[126] E. M. V. Reiche , S. O. V. Nunes , and H. K. Morimoto , “Stress, Depression, the Immune System, and Cancer,” Lancet Oncology 5, no. 10 (2004): 617–625, https://www.thelancet.com/journals/lanonc/article/PIIS1470‐2045(04)01597‐9/abstract. 15465465 10.1016/S1470-2045(04)01597-9

[127] S. M. Smith and W. W. Vale , “The Role of the Hypothalamic‐Pituitary‐Adrenal Axis in Neuroendocrine Responses to Stress,” Dialogues in Clinical Neuroscience 8 (2024): 383–395, 10.31887/DCNS.2006.8.4/ssmith. PMC3181830 17290797

[128] S. E. Sephton , F. S. Dhabhar , A. S. Keuroghlian , et al., “Depression, Cortisol, and Suppressed Cell‐Mediated Immunity in Metastatic Breast Cancer,” Brain, Behavior, and Immunity 23, no. 8 (November 2009): 1148–1155. 19643176 10.1016/j.bbi.2009.07.007

[129] A. Z. Weinrib , S. E. Sephton , K. DeGeest , et al., “Diurnal Cortisol Dysregulation, Functional Disability, and Depression in Women With Ovarian Cancer,” Cancer 116, no. 18 (2010): 4410–4419, 10.1002/cncr.25299. 20564155 PMC3118555

[130] A. K. Singh , U. Chatterjee , C. R. MacDonald , E. A. Repasky , and U. Halbreich , “Psychosocial Stress and Immunosuppression in Cancer: What Can We Learn From New Research?,” BJPsych Advances 27, no. 3 (May 2021): 187–197, https://www.cambridge.org/core/journals/bjpsych‐advances/article/psychosocial‐stress‐and‐immunosuppression‐in‐cancer‐what‐can‐we‐learn‐from‐new‐research/E5B099FB02343C2FAA8F7170B3933B5C. 34295535 10.1192/bja.2021.9 PMC8294471

[131] M. B. Currier and C. B. Nemeroff , “Depression as a Risk Factor for Cancer: From Pathophysiological Advances to Treatment Implications,” Annual Review of Medicine 65 (January 2014): 203–221, 10.1146/annurev-med-061212-171507. 24215332

[132] Z. Kronfol , “Immune Dysregulation in Major Depression: A Critical Review of Existing Evidence,” International Journal of Neuropsychopharmacology 5, no. 4 (December 2002): 333–343, 10.1017/S1461145702003024. 12466033

[133] S. K. Budania , M. Rathi , S. Singh , and S. Yadav , “Depression and Cancer: An Update,” Journal of the Scientific Society 41, no. 3 (December 2014): 232, https://journals.lww.com/jsci/fulltext/2014/41030/depression_and_cancer__an_update.23.aspx.

[134] B. L. Andersen , J. Myers , T. Blevins , et al., “Depression in Association With Neutrophil‐To‐Lymphocyte, Platelet‐To‐Lymphocyte, and Advanced Lung Cancer Inflammation Index Biomarkers Predicting Lung Cancer Survival,” PLoS One 18 (February 2023): e0282206, 10.1371/journal.pone.0282206. 36827396 PMC9956881

[135] F. S. Barreto , A. J. M. Chaves Filho , M. C. C. R. de Araújo , et al., “Tryptophan Catabolites Along the Indoleamine 2,3‐Dioxygenase Pathway as a Biological Link Between Depression and Cancer,” Behavioural Pharmacology 29 (April 2018): 165–180. 29543650 10.1097/FBP.0000000000000384

[136] D. L. Kelly , D. E. Lyon , S. L. Yoon , and A. L. Horgas , “The Microbiome and Cancer: Implications for Oncology Nursing Science,” Cancer Nursing 39, no. 3 (June 2016): E56, https://journals.lww.com/cancernursingonline/fulltext/2016/05000/the_microbiome_and_cancer__implications_for.19.aspx. 26110573 10.1097/NCC.0000000000000286

[137] N. A. Amran , M. H. Arzmi , A. F. I. Awang , et al., “Microbiome Dysbiosis in Depression: A Narrative Review,” IIUM Medical Journal Malaysia 23, no. 3 (July 2024): 1–22, https://journals.iium.edu.my/kom/index.php/imjm/article/view/2293.

[138] M. Severo , A. Ventriglio , D. Bhugra , and A. Petito , “Psychobiological Screening Among Patients Affected by Prostate Cancer: Identification of Potential Psychobiological Markers,” Industrial Psychiatry Journal 32, no. Suppl 1 (November 2023): S273, https://journals.lww.com/inpj/fulltext/2023/32001/psychobiological_screening_among_patients_affected.49.aspx. 38370936 10.4103/ipj.ipj_212_23 PMC10871439

[139] F. J. McMahon , “Clinically Useful Genetic Markers of Antidepressant Response: How Do We Get There From Here?,” AJP 172, no. 8 (August 2015): 697–699, 10.1176/appi.ajp.2015.15050644. 26234589

[140] J. M. Kim , H. J. Kang , S. Y. Kim , et al., “BDNF Promoter Methylation Associated With Suicidal Ideation in Patients With Breast Cancer,” International Journal of Psychiatry in Medicine 49, no. 1 (January 2015): 75–94, 10.1177/0091217415574439. 25838322

[141] C. U. Pae , A. Serretti , L. Mandelli , et al., “Dysbindin Associated With Selective Serotonin Reuptake Inhibitor Antidepressant Efficacy,” Pharmacogenetics and Genomics 17 (January 2007): 69–75, https://journals.lww.com/jpharmacogenetics/abstract/2007/01000/dysbindin_associated_with_selective_serotonin.7.aspx. 17264804 10.1097/01.fpc.0000236330.03681.6d

[142] M. B. Abubakar and S. H. Gan , “Genetic Polymorphisms Associated With Common Symptoms Experienced by Breast Cancer Patients,” Biomedical Research 29, no. 6 (2018): 1164–1175.

[143] J. H. Lee , I. Hwang , Y. N. Kang , I. J. Choi , and D. K. Kim , “Genetic Characteristics of Mitochondrial DNA Was Associated With Colorectal Carcinogenesis and Its Prognosis,” PLoS One 10 (March 2015): e0118612, 10.1371/journal.pone.0118612. 25734426 PMC4348484

[144] K. Litzelman and M. Verma , “Epigenetic Regulation in Biopsychosocial Pathways,” Methods in Molecular Biology 1238 (2015): 549–567. 25421680 10.1007/978-1-4939-1804-1_29

[145] M. Verma , S. Rogers , R. L. Divi , et al., “Epigenetic Research in Cancer Epidemiology: Trends, Opportunities, and Challenges,” Cancer Epidemiology, Biomarkers & Prevention 23, no. 2 (February 2014): 223–233. 10.1158/1055-9965.EPI-13-0573 PMC3925982 24326628

[146] A. P. Feinberg , “The Epigenetics of Cancer Etiology,” Seminars in Cancer Biology 14, no. 6 (December 2004): 427–432. 15489135 10.1016/j.semcancer.2004.06.005

[147] M. Kelly‐Irving , L. Mabile , P. Grosclaude , T. Lang , and C. Delpierre , “The Embodiment of Adverse Childhood Experiences and Cancer Development: Potential Biological Mechanisms and Pathways Across the Life Course,” International Journal of Public Health 58, no. 1 (February 2013): 3–11. 22588310 10.1007/s00038-012-0370-0

[148] H. Hong , M. Ji , and D. Lai , “Chronic Stress Effects on Tumor: Pathway and Mechanism,” Frontiers in Oncology 11 (2021): 738252. 34988010 10.3389/fonc.2021.738252 PMC8720973

[149] S. Patnaik and Anupriya , “Drugs Targeting Epigenetic Modifications and Plausible Therapeutic Strategies Against Colorectal Cancer,” Frontiers in Pharmacology 10 (June 2019): 588: 1–15. 10.3389/fphar.2019.00588/full. 31244652 PMC6563763

[150] P. M. Biava and G. Norbiato , “Getting an Insight Into the Complexity of Major Chronic Inflammatory and Degenerative Diseases: A Potential New Systemic Approach to Their Treatment,” 2024, https://www.eurekaselect.com/article/68854. 10.2174/138920101609150715141308 26201608

[151] M. Huo , J. Zhang , W. Huang , and Y. Wang , “Interplay Among Metabolism, Epigenetic Modifications, and Gene Expression in Cancer,” Frontiers in Cell and Developmental Biology 9 (December 2021): 793428, 10.3389/fcell.2021.793428/full. 35004688 PMC8740611

[152] M. Widschwendter and P. A. Jones , “DNA Methylation and Breast Carcinogenesis,” Oncogene 21, no. 35 (August 2002): 5462–5482, https://www.nature.com/articles/1205606. 12154408 10.1038/sj.onc.1205606

[153] B. Dell'Osso , C. D'Addario , M. Carlotta Palazzo , et al., “Epigenetic Modulation of BDNF Gene: Differences in DNA Methylation Between Unipolar and Bipolar Patients,” Journal of Affective Disorders 166 (September 2014): 330–333, https://www.sciencedirect.com/science/article/pii/S0165032714003085. 25012449 10.1016/j.jad.2014.05.020

[154] M. Kundakovic , K. Gudsnuk , J. B. Herbstman , D. Tang , F. P. Perera , and F. A. Champagne , “DNA Methylation of BDNF as a Biomarker of Early‐Life Adversity,” Proceedings of the National Academy of Sciences 112, no. 22 (June 2015): 6807–6813, 10.1073/pnas.1408355111. PMC4460453 25385582

[155] S. I. Deutsch , R. B. Rosse , J. Mastropaolo , K. D. Long , and B. L. Gaskins , “Epigenetic Therapeutic Strategies for the Treatment of Neuropsychiatric Disorders: Ready for Prime Time?,” Clinical Neuropharmacology 2 (April 2008): 104, https://journals.lww.com/clinicalneuropharm/abstract/2008/03000/epigenetic_therapeutic_strategies_for_the.7.aspx. 10.1097/WNF.0b013e318067e255 18382183

[156] A. Wang , “Understanding the Role of Epigenetics in Adverse Health Outcomes,” Journal of Student Research 12, no. 4 (November 2023): 1–3, https://www.jsr.org/hs/index.php/path/article/view/5524.

[157] I. C. G. Weaver , N. Cervoni , F. A. Champagne , et al., “Epigenetic Programming by Maternal Behavior,” Nature Neuroscience 7, no. 8 (August 2004): 847–854. 15220929 10.1038/nn1276

[158] D. K. Sarkar , “Male Germline Transmits Fetal Alcohol Epigenetic Marks for Multiple Generations: A Review,” Addiction Biology 21, no. 1 (2016): 23–34, 10.1111/adb.12186. 25581210 PMC7250160

[159] C. Pu , S. Tian , S. He , et al., “Depression and Stress Levels Increase Risk of Liver Cancer Through Epigenetic Downregulation of Hypocretin,” Genes and Diseases 9, no. 4 (July 2022): 1024–1037. 35685472 10.1016/j.gendis.2020.11.013 PMC9170575

[160] M. Sachdeva and Y. Y. Mo , “miR‐145‐Mediated Suppression of Cell Growth, Invasion and Metastasis,” American Journal of Translational Research 2, no. 2 (March 2010): 170–180. 20407606 PMC2855636

[161] D. F. Quail and J. A. Joyce , “Microenvironmental Regulation of Tumor Progression and Metastasis,” Nature Medicine 19, no. 11 (November 2013): 1423–1437. 10.1038/nm.3394 PMC3954707 24202395

[162] M. Batie , J. Frost , M. Frost , J. W. Wilson , P. Schofield , and S. Rocha , “Hypoxia Induces Rapid Changes to Histone Methylation and Reprograms Chromatin,” Science 363, no. 6432 (March 2019): 1222–1226. 30872526 10.1126/science.aau5870

[163] D. Ravindran Menon , H. Hammerlindl , J. Torrano , H. Schaider , and M. Fujita , “Epigenetics and Metabolism at the Crossroads of Stress‐Induced Plasticity, Stemness and Therapeutic Resistance in Cancer,” Theranostics 10, no. 14 (2020): 6261–6277. 32483452 10.7150/thno.42523 PMC7255038

[164] L. Colyn , E. Venzala , S. Marco , I. Pérez‐Otaño , and R. Tordera , “Chronic Social Defeat Stress Induces Sustained Synaptic Structural Changes in the Prefrontal Cortex and Amygdala,” Behavioural Brain Research 1, no. 373 (July 2019): 112079. 10.1016/j.bbr.2019.112079 31301411

[165] M. D. Weber , J. P. Godbout , and J. F. Sheridan , “Repeated Social Defeat, Neuroinflammation, and Behavior: Monocytes Carry the Signal,” Neuropsychopharmacology 42, no. 1 (January 2017): 46–61. 27319971 10.1038/npp.2016.102 PMC5143478

[166] E. S. Wohleb , D. B. McKim , J. F. Sheridan , and J. P. Godbout , “Monocyte Trafficking to the Brain With Stress and Inflammation: A Novel Axis of Immune‐to‐Brain Communication That Influences Mood and Behavior,” Frontiers in Neuroscience 8 (2014): 447. 25653581 10.3389/fnins.2014.00447 PMC4300916

[167] D. McCaffrey , A. J. Lawther , C. S. Weickert , and A. K. Walker , “Cancer Activates Microglia to the Same Extent as Chronic Stress Throughout Stress Neurocircuitry in a Mouse Model of Breast Cancer,” Psychoneuroendocrinology 146 (December 2022): 105938. 36174451 10.1016/j.psyneuen.2022.105938

[168] K. Ramirez , J. Fornaguera‐Trías , and J. F. Sheridan , “Stress‐Induced Microglia Activation and Monocyte Trafficking to the Brain Underlie the Development of Anxiety and Depression,” Current Topics in Behavioral Neurosciences 31 (2017): 155–172. 27352390 10.1007/7854_2016_25

[169] X. Feng , Y. Zhao , T. Yang , et al., “Glucocorticoid‐Driven NLRP3 Inflammasome Activation in Hippocampal Microglia Mediates Chronic Stress‐Induced Depressive‐Like Behaviors,” Frontiers in Molecular Neuroscience 12 (2019): 210. 31555091 10.3389/fnmol.2019.00210 PMC6727781

[170] E. Schramm and A. Waisman , “Microglia as Central Protagonists in the Chronic Stress Response,” Neuroimmunology and Neuroinflammation 9, no. 6 (November 2022): e200023. 36357946 10.1212/NXI.0000000000200023 PMC9453699

[171] S. Dai , Y. Mo , Y. Wang , et al., “Chronic Stress Promotes Cancer Development,” Frontiers in Oncology 10 (August 2020): 1492, 10.3389/fonc.2020.01492/full. 32974180 PMC7466429

[172] Z. Feng , L. Liu , C. Zhang , et al., “Chronic Restraint Stress Attenuates p53 Function and Promotes Tumorigenesis,” Proceedings of the National Academy of Sciences of the United States of America 109, no. 18 (May 2012): 7013–7018. 22509031 10.1073/pnas.1203930109 PMC3345015

[173] L. Zong , S. Mo , Z. Sun , et al., “Analysis of the Immune Checkpoint V‐Domain Ig‐Containing Suppressor of T‐Cell Activation (VISTA) in Endometrial Cancer,” Modern Pathology 35, no. 2 (February 2022): 266–273. 34493823 10.1038/s41379-021-00901-y

[174] T. Heidt , H. B. Sager , G. Courties , et al., “Chronic Variable Stress Activates Hematopoietic Stem Cells,” Nature Medicine 20, no. 7 (July 2014): 754–758. 10.1038/nm.3589 PMC4087061 24952646

[175] S. Vignjević Petrinović , M. Budeč , D. Marković , et al., “Nitric Oxide‐Dependent Expansion of Erythroid Progenitors in a Murine Model of Chronic Psychological Stress,” Histochemistry and Cell Biology 153, no. 6 (June 2020): 457–468. 32144481 10.1007/s00418-020-01856-y

[176] J. Tower , “Stress and Stem Cells,” Wiley Interdisciplinary Reviews: Developmental Biology 1, no. 6 (2012): 789–802. 23799624 10.1002/wdev.56 PMC3812933

[177] E. Chad‐Friedman , S. Coleman , L. N. Traeger , et al., “Psychological Distress Associated With Cancer Screening: A Systematic Review,” Cancer 123, no. 20 (October 2017): 3882–3894. 28833054 10.1002/cncr.30904 PMC8116666

[178] M. H. Antoni and F. S. Dhabhar , “The Impact of Psychosocial Stress and Stress Management on Immune Responses in Patients With Cancer,” Cancer 125, no. 9 (May 2019): 1417–1431. 30768779 10.1002/cncr.31943 PMC6467795

[179] X. Wang , N. Wang , L. Zhong , et al., “Prognostic Value of Depression and Anxiety on Breast Cancer Recurrence and Mortality: A Systematic Review and Meta‐Analysis of 282,203 Patients,” Molecular Psychiatry 25, no. 12 (December 2020): 3186–3197. 32820237 10.1038/s41380-020-00865-6 PMC7714689

[180] S. L. Kim , H. S. Choi , J. H. Kim , and D. S. Lee , “The Antiasthma Medication Ciclesonide Suppresses Breast Cancer Stem Cells Through Inhibition of the Glucocorticoid Receptor Signaling‐Dependent YAP Pathway,” Molecules 25, no. 24 (December 2020): 6028. 33352739 10.3390/molecules25246028 PMC7766992

[181] S. Khadka , S. R. Druffner , B. C. Duncan , and J. T. Busada , “Glucocorticoid Regulation of Cancer Development and Progression,” Frontiers in Endocrinology (Lausanne) 14 (2023): 1161768. 10.3389/fendo.2023.1161768 PMC10151568 37143725

[182] H. M. Liu , L. le Ma , C. Li , et al., “The Molecular Mechanism of Chronic Stress Affecting the Occurrence and Development of Breast Cancer and Potential Drug Therapy,” Translational Oncology 15, no. 1 (January 2022): 101281. 34875482 10.1016/j.tranon.2021.101281 PMC8652015

[183] L. He , L. Yuan , Y. Sun , et al., “Glucocorticoid Receptor Signaling Activates TEAD4 to Promote Breast Cancer Progression,” Cancer Research 79, no. 17 (September 2019): 4399–4411. 31289134 10.1158/0008-5472.CAN-19-0012

[184] E. S. Miller , C. G. Apple , K. B. Kannan , et al., “Chronic Stress Induces Persistent Low‐Grade Inflammation,” American Journal of Surgery 218, no. 4 (October 2019): 677–683. 31378316 10.1016/j.amjsurg.2019.07.006 PMC6768696

[185] N. Rohleder , “Stimulation of Systemic Low‐Grade Inflammation by Psychosocial Stress,” Psychosomatic Medicine 76, no. 3 (April 2014): 181–189. 24608036 10.1097/PSY.0000000000000049

[186] G. D'Orazi , M. Cordani , and M. Cirone , “Oncogenic Pathways Activated by Pro‐Inflammatory Cytokines Promote Mutant p53 Stability: Clue for Novel Anticancer Therapies,” Cellular and Molecular Life Sciences 78, no. 5 (March 2021): 1853–1860. 33070220 10.1007/s00018-020-03677-7 PMC11072129

[187] Y. Choi , J. K. Kim , and J. Y. Yoo , “NFκB and STAT3 Synergistically Activate the Expression of FAT10, a Gene Counteracting the Tumor Suppressor p53,” Molecular Oncology 8, no. 3 (May 2014): 642–655. 24518302 10.1016/j.molonc.2014.01.007 PMC5528625

[188] M. A. Romeo , M. S. Gilardini Montani , R. Benedetti , A. Arena , G. D'Orazi , and M. Cirone , “p53‐R273H Sustains ROS, Pro‐Inflammatory Cytokine Release and mTOR Activation While Reducing Autophagy, Mitophagy and UCP2 Expression, Effects Prevented by wtp53,” Biomolecules 11, no. 3 (February 2021): 344. 33668399 10.3390/biom11030344 PMC7996167

[189] A. E. R. Kartikasari , C. S. Huertas , A. Mitchell , and M. Plebanski , “Tumor‐Induced Inflammatory Cytokines and the Emerging Diagnostic Devices for Cancer Detection and Prognosis,” Frontiers in Oncology 11 (2021): 692142. 34307156 10.3389/fonc.2021.692142 PMC8294036

[190] Y. Sun , Y. Koyama , and S. Shimada , “Inflammation From Peripheral Organs to the Brain: How Does Systemic Inflammation Cause Neuroinflammation?,” Frontiers in Aging Neuroscience 14 (2022): 903455. 35783147 10.3389/fnagi.2022.903455 PMC9244793

[191] M. Maes , “Evidence for an Immune Response in Major Depression: A Review and Hypothesis,” Progress in Neuro‐Psychopharmacology & Biological Psychiatry 19, no. 1 (January 1995): 11–38. 7708925 10.1016/0278-5846(94)00101-m

[192] M. Maes , H. Y. Meltzer , E. Bosmans , et al., “Increased Plasma Concentrations of Interleukin‐6, Soluble Interleukin‐6, Soluble Interleukin‐2 and Transferrin Receptor in Major Depression,” Journal of Affective Disorders 34, no. 4 (August 1995): 301–309. 8550956 10.1016/0165-0327(95)00028-l

[193] B. Polityńska , O. Pokorska , A. M. Wojtukiewicz , et al., “Is Depression the Missing Link Between Inflammatory Mediators and Cancer?,” Pharmacology & Therapeutics 240 (December 2022): 108293. 36216210 10.1016/j.pharmthera.2022.108293

[194] W. Shi‐Heng , L. Y. Hsu , M. C. Lin , and C. S. Wu , “Associations Between Depression and Cancer Risk Among Patients With Diabetes Mellitus: A Population‐Based Cohort Study,” Cancer Medicine 12, no. 19 (October 2023): 19968–19977. 37706606 10.1002/cam4.6539 PMC10587979

[195] K. Ohi , “Shared Genetic Correlation and Causal Association Between Major Depressive Disorder and Breast Cancer,” BMC Medicine 21, no. 1 (June 2023): 203. 37280618 10.1186/s12916-023-02905-8 PMC10242811

[196] X. Wu , W. Zhang , X. Zhao , et al., “Investigating the Relationship Between Depression and Breast Cancer: Observational and Genetic Analyses,” BMC Medicine 21, no. 1 (May 2023): 170. 37143087 10.1186/s12916-023-02876-w PMC10161423

[197] Y. Lu , Y. Qi , J. Du , et al., “Classification of High‐Risk Depressed Mood Groups in Cancer Patients Based on Health Ecology Model,” Journal of Affective Disorders 15, no. 347 (February 2024): 327–334. 10.1016/j.jad.2023.11.061 37992777

[198] L. M. Pyter , V. Pineros , J. A. Galang , M. K. McClintock , and B. J. Prendergast , “Peripheral Tumors Induce Depressive‐Like Behaviors and Cytokine Production and Alter Hypothalamic‐Pituitary‐Adrenal Axis Regulation,” Proceedings of the National Academy of Sciences of the United States of America 106, no. 22 (June 2009): 9069–9074. 19451634 10.1073/pnas.0811949106 PMC2689998

[199] A. Gartung , J. Yang , V. P. Sukhatme , et al., “Suppression of Chemotherapy‐Induced Cytokine/Lipid Mediator Surge and Ovarian Cancer by a Dual COX‐2/sEH Inhibitor,” Proceedings of the National Academy of Sciences of the United States of America 116, no. 5 (January 2019): 1698–1703. 30647111 10.1073/pnas.1803999116 PMC6358686

[200] C. A. Barker , S. K. Kim , S. Budhu , K. Matsoukas , A. F. Daniyan , and S. P. D'Angelo , “Cytokine Release Syndrome After Radiation Therapy: Case Report and Review of the Literature,” Journal for Immunotherapy of Cancer 6, no. 1 (January 2018): 1. 29298730 10.1186/s40425-017-0311-9 PMC5795275

[201] S. G. Smith , K. Smits , S. A. Joosten , et al., “Intracellular Cytokine Staining and Flow Cytometry: Considerations for Application in Clinical Trials of Novel Tuberculosis Vaccines,” PLoS One 10, no. 9 (2015): e0138042. 26367374 10.1371/journal.pone.0138042 PMC4569436

[202] L. Grassi , M. G. Nanni , G. Rodin , M. Li , and R. Caruso , “The Use of Antidepressants in Oncology: A Review and Practical Tips for Oncologists,” Annals of Oncology 29, no. 1 (January 2018): 101–111. 29272358 10.1093/annonc/mdx526

[203] Z. G. Laoutidis and K. Mathiak , “Antidepressants in the Treatment of Depression/Depressive Symptoms in Cancer Patients: A Systematic Review and Meta‐Analysis,” BMC Psychiatry 13, no. 1 (May 2013): 140, 10.1186/1471-244X-13-140. 23679841 PMC3674917

[204] G. Vita , B. Compri , F. Matcham , C. Barbui , and G. Ostuzzi , “Antidepressants for the Treatment of Depression in People With Cancer—Vita, G,” 2023, Cochrane Library, 10.1002/14651858.CD011006.pub4/full. PMC10065046 36999619

[205] S. Zaini , N. C. Guan , A. H. Sulaiman , N. Z. Zainal , H. Z. Huri , and S. H. Shamsudin , “The Use of Antidepressants for Physical and Psychological Symptoms in Cancer,” Current Drug Targets 19, no. 12 (2018): 1431–1455. 29484993 10.2174/1389450119666180226125026

[206] G. Ostuzzi , F. Matcham , S. Dauchy , C. Barbui , and M. Hotopf , “Antidepressants for the Treatment of Depression in People With Cancer—Ostuzzi, G,” 2018, Cochrane Library, 10.1002/14651858.CD011006.pub3/full. PMC6494588 29683474

[207] Y. Song , X. Yang , and B. Yu , “Repurposing Antidepressants for Anticancer Drug Discovery,” Drug Discovery Today 27, no. 7 (July 2022): 1924–1935, https://www.sciencedirect.com/science/article/pii/S135964462100475X. 34728374 10.1016/j.drudis.2021.10.019

[208] Y. Zheng , X. Chang , Y. Huang , and D. He , “The Application of Antidepressant Drugs in Cancer Treatment,” Biomedicine & Pharmacotherapy 157 (January 2023): 113985, https://www.sciencedirect.com/science/article/pii/S0753332222013749. 36402031 10.1016/j.biopha.2022.113985

[209] B. Abadi , Y. Shahsavani , M. Faramarzpour , N. Rezaei , and H. R. Rahimi , “Antidepressants With Anti‐Tumor Potential in Treating Glioblastoma: A Narrative Review,” Fundamental & Clinical Pharmacology 36, no. 1 (February 2022): 35–48. 34212424 10.1111/fcp.12712

[210] K. Hu , A. Sjölander , D. Lu , et al., “Aspirin and Other Non‐Steroidal Anti‐Inflammatory Drugs and Depression, Anxiety, and Stress‐Related Disorders Following a Cancer Diagnosis: A Nationwide Register‐Based Cohort Study,” BMC Medicine 18, no. 1 (September 2020): 238. 32900363 10.1186/s12916-020-01709-4 PMC7487710

[211] N. F. Shaikh , C. Shen , T. LeMasters , N. Dwibedi , A. Ladani , and U. Sambamoorthi , “Prescription Non‐Steroidal Anti‐Inflammatory Drugs (NSAIDs) and Incidence of Depression Among Older Cancer Survivors With Osteoarthritis: A Machine Learning Analysis,” Cancer Informatics 22 (2023): 11769351231165161. 37101728 10.1177/11769351231165161 PMC10123903

[212] L. Rayner , A. Price , A. Evans , K. Valsraj , I. J. Higginson , and M. Hotopf , “Antidepressants for Depression in Physically Ill People,” Cochrane Database of Systematic Reviews 17, no. 3 (March 2010): CD007503. 10.1002/14651858.CD007503.pub2 PMC12279289 20238354

[213] R. Sloman , “Relaxation and Imagery for Anxiety and Depression Control in Community Patients With Advanced Cancer,” Cancer Nursing 25, no. 6 (December 2002): 432–435. 12464834 10.1097/00002820-200212000-00005

[214] D. L. Feros , L. Lane , J. Ciarrochi , and J. T. Blackledge , “Acceptance and Commitment Therapy (ACT) for Improving the Lives of Cancer Patients: A Preliminary Study,” Psycho‐Oncology 22, no. 2 (February 2013): 459–464. 23382134 10.1002/pon.2083

[215] L. L. Craft , E. H. Vaniterson , I. B. Helenowski , A. W. Rademaker , and K. S. Courneya , “Exercise Effects on Depressive Symptoms in Cancer Survivors: A Systematic Review and Meta‐Analysis,” Cancer Epidemiology, Biomarkers & Prevention 21, no. 1 (January 2012): 3–19. 10.1158/1055-9965.EPI-11-0634 PMC3253916 22068286

[216] L. E. Carlson , T. L. Beattie , J. Giese‐Davis , et al., “Mindfulness‐Based Cancer Recovery and Supportive‐Expressive Therapy Maintain Telomere Length Relative to Controls in Distressed Breast Cancer Survivors,” Cancer 121, no. 3 (Febraury 2015): 476–484. 25367403 10.1002/cncr.29063

[217] B. A. McGregor , M. H. Antoni , A. Boyers , S. M. Alferi , B. B. Blomberg , and C. S. Carver , “Cognitive‐Behavioral Stress Management Increases Benefit Finding and Immune Function Among Women With Early‐Stage Breast Cancer,” Journal of Psychosomatic Research 56, no. 1 (January 2004): 1–8. 14987957 10.1016/S0022-3999(03)00036-9

[218] B. L. Andersen , W. B. Farrar , D. M. Golden‐Kreutz , et al., “Psychological, Behavioral, and Immune Changes After a Psychological Intervention: A Clinical Trial,” Journal of Clinical Oncology 22, no. 17 (September 2004): 3570–3580. 15337807 10.1200/JCO.2004.06.030 PMC2168591

[219] O. Eremin , M. B. Walker , E. Simpson , et al., “Immuno‐Modulatory Effects of Relaxation Training and Guided Imagery in Women With Locally Advanced Breast Cancer Undergoing Multimodality Therapy: A Randomised Controlled Trial,” Breast 18, no. 1 (February 2009): 17–25. 19008099 10.1016/j.breast.2008.09.002

[220] T. Hanalis‐Miller , I. Ricon‐Becker , N. Sakis , et al., “Peri‐Operative Individually Tailored Psychological Intervention in Breast Cancer Patients Improves Psychological Indices and Molecular Biomarkers of Metastasis in Excised Tumors,” Brain, Behavior, and Immunity 117 (March 2024): 529–540. 38346596 10.1016/j.bbi.2024.02.009

[221] D. H. Kang , T. McArdle , N. J. Park , M. T. Weaver , B. Smith , and J. Carpenter , “Dose Effects of Relaxation Practice on Immune Responses in Women Newly Diagnosed With Breast Cancer: An Exploratory Study,” Oncology Nursing Forum 38, no. 3 (May 2011): E240–E252. 21531674 10.1188/11.ONF.E240-E252

[222] L. Cohen , P. A. Parker , L. Vence , et al., “Presurgical Stress Management Improves Postoperative Immune Function in Men With Prostate Cancer Undergoing Radical Prostatectomy,” Psychosomatic Medicine 73, no. 3 (April 2011): 218–225. 21257977 10.1097/PSY.0b013e31820a1c26

[223] M. F. Zhang , Y. S. Wen , W. Y. Liu , L. F. Peng , X. D. Wu , and Q. W. Liu , “Effectiveness of Mindfulness‐Based Therapy for Reducing Anxiety and Depression in Patients With Cancer: A Meta‐Analysis,” Medicine 94, no. 45 (November 2015): e0897, https://www.ncbi.nlm.nih.gov/pmc/articles/PMC4912240/. 26559246 10.1097/MD.0000000000000897 PMC4912240

[224] K. M. Phillips , M. H. Antoni , S. C. Lechner , et al., “Stress Management Intervention Reduces Serum Cortisol and Increases Relaxation During Treatment for Nonmetastatic Breast Cancer,” Psychosomatic Medicine 70, no. 9 (November 2008): 1044–1049. 18842742 10.1097/PSY.0b013e318186fb27 PMC5761725

[225] F. J. Penedo , R. S. Fox , E. A. Walsh , et al., “Effects of Web‐Based Cognitive Behavioral Stress Management and Health Promotion Interventions on Neuroendocrine and Inflammatory Markers in Men With Advanced Prostate Cancer: A Randomized Controlled Trial,” Brain, Behavior, and Immunity 95 (July 2021): 168–177. 33737170 10.1016/j.bbi.2021.03.014 PMC8888023

[226] M. H. Antoni , S. Lechner , A. Diaz , et al., “Cognitive Behavioral Stress Management Effects on Psychosocial and Physiological Adaptation in Women Undergoing Treatment for Breast Cancer,” Brain, Behavior, and Immunity 23, no. 5 (July 2009): 580–591. 18835434 10.1016/j.bbi.2008.09.005 PMC2722111

[227] K. Sanada , M. Alda Díez , M. Salas Valero , et al., “Effects of Mindfulness‐Based Interventions on Biomarkers in Healthy and Cancer Populations: A Systematic Review,” BMC Complementary and Alternative Medicine 17, no. 1 (February 2017): 125. 28231775 10.1186/s12906-017-1638-y PMC5324275

[228] C. A. Lengacher , R. R. Reich , C. L. Paterson , et al., “A Large Randomized Trial: Effects of Mindfulness‐Based Stress Reduction (MBSR) for Breast Cancer (BC) Survivors on Salivary Cortisol and IL‐6,” Biological Research for Nursing 21, no. 1 (January 2019): 39–49. 30079756 10.1177/1099800418789777 PMC6700883

[229] M. Warth , F. Koehler , C. Aguilar‐Raab , H. J. Bardenheuer , B. Ditzen , and J. Kessler , “Stress‐Reducing Effects of a Brief Mindfulness Intervention in Palliative Care: Results From a Randomised, Crossover Study,” European Journal of Cancer Care 29, no. 4 (July 2020): e13249. 32436277 10.1111/ecc.13249

[230] H. Park , S. Oh , Y. Noh , J. Y. Kim , and J. H. Kim , “Heart Rate Variability as a Marker of Distress and Recovery: The Effect of Brief Supportive Expressive Group Therapy With Mindfulness in Cancer Patients,” Integrative Cancer Therapies 17, no. 3 (September 2018): 825–831. 29417836 10.1177/1534735418756192 PMC6142099

[231] A. Matiz , B. Scaggiante , C. Conversano , et al., “The Effect of Mindfulness‐Based Interventions on Biomarkers in Cancer Patients and Survivors: A Systematic Review,” Stress and Health 40, no. 4 (August 2024): e3375. 38259050 10.1002/smi.3375

[232] E. Järvelä‐Reijonen , S. Puttonen , L. Karhunen , et al., “The Effects of Acceptance and Commitment Therapy (ACT) Intervention on Inflammation and Stress Biomarkers: A Randomized Controlled Trial,” International Journal of Behavioral Medicine 27, no. 5 (October 2020): 539–555, 10.1007/s12529-020-09891-8. 32394219 PMC7497453

[233] R. M. Rao , H. R. Nagendra , N. Raghuram , et al., “Influence of Yoga on Mood States, Distress, Quality of Life and Immune Outcomes in Early Stage Breast Cancer Patients Undergoing Surgery,” International Journal of Yoga 1, no. 1 (January 2008): 11–20. 21829279 10.4103/0973-6131.36789 PMC3144603

[234] H. S. Vadiraja , R. M. Raghavendra , R. Nagarathna , et al., “Effects of a Yoga Program on Cortisol Rhythm and Mood States in Early Breast Cancer Patients Undergoing Adjuvant Radiotherapy: A Randomized Controlled Trial,” Integrative Cancer Therapies 8, no. 1 (March 2009): 37–46. 19190034 10.1177/1534735409331456

[235] M. E. Schmidt , A. Meynköhn , N. Habermann , et al., “Resistance Exercise and Inflammation in Breast Cancer Patients Undergoing Adjuvant Radiation Therapy: Mediation Analysis From a Randomized, Controlled Intervention Trial,” International Journal of Radiation Oncology, Biology, Physics 94, no. 2 (February 2016): 329–337. 26853341 10.1016/j.ijrobp.2015.10.058

[236] L. Q. Rogers , E. McAuley , P. M. Anton , et al., “Better Exercise Adherence After Treatment for Cancer (BEAT Cancer) Study: Rationale, Design, and Methods,” Contemporary Clinical Trials 33, no. 1 (January 2012): 124–137. 21983625 10.1016/j.cct.2011.09.004 PMC3253876

[237] E. Scott , A. J. Daley , H. Doll , et al., “Effects of an Exercise and Hypocaloric Healthy Eating Program on Biomarkers Associated With Long‐Term Prognosis After Early‐Stage Breast Cancer: A Randomized Controlled Trial,” Cancer Causes & Control 24, no. 1 (2013): 181–191. 23184120 10.1007/s10552-012-0104-x

[238] C. A. Befort , J. R. Klemp , H. L. Austin , et al., “Outcomes of a Weight Loss Intervention Among Rural Breast Cancer Survivors,” Breast Cancer Research and Treatment 132, no. 2 (2012): 631–639. 22198470 10.1007/s10549-011-1922-3 PMC3314288

[239] A. D. Hagstrom , P. W. M. Marshall , C. Lonsdale , et al., “The Effect of Resistance Training on Markers of Immune Function and Inflammation in Previously Sedentary Women Recovering From Breast Cancer: A Randomized Controlled Trial,” Breast Cancer Research and Treatment 155, no. 3 (February 2016): 471–482. 26820653 10.1007/s10549-016-3688-0

[240] B. Oh , P. Butow , B. Mullan , et al., “Impact of Medical Qigong on Quality of Life, Fatigue, Mood and Inflammation in Cancer Patients: A Randomized Controlled Trial,” Annals of Oncology 21, no. 3 (March 2010): 608–614. 19880433 10.1093/annonc/mdp479 PMC2826100

[241] F. H. Hsiao , G. M. Jow , W. H. Kuo , et al., “The Effects of Psychotherapy on Psychological Well‐Being and Diurnal Cortisol Patterns in Breast Cancer Survivors,” Psychotherapy and Psychosomatics 81, no. 3 (2012): 173–182. 22399076 10.1159/000329178

[242] M. Hernandez‐Reif , G. Ironson , T. Field , et al., “Breast Cancer Patients Have Improved Immune and Neuroendocrine Functions Following Massage Therapy,” Journal of Psychosomatic Research 57, no. 1 (July 2004): 45–52. 15256294 10.1016/S0022-3999(03)00500-2

[243] I. Osaka , Y. Kurihara , K. Tanaka , H. Nishizaki , S. Aoki , and I. Adachi , “Endocrinological Evaluations of Brief Hand Massages in Palliative Care,” Journal of Alternative and Complementary Medicine 15, no. 9 (September 2009): 981–985. 19757975 10.1089/acm.2008.0241

[244] M. Warth , J. Kessler , S. Kotz , T. K. Hillecke , and H. J. Bardenheuer , “Effects of Vibroacoustic Stimulation in Music Therapy for Palliative Care Patients: A Feasibility Study,” BMC Complementary and Alternative Medicine 15, no. 1 (December 2015): 436, 10.1186/s12906-015-0933-8. 26669437 PMC4681146

[245] F. Koehler , J. Kessler , M. Stoffel , et al., “Psychoneuroendocrinological Effects of Music Therapy Versus Mindfulness in Palliative Care: Results From the ‘Song of Life’ Randomized Controlled Trial,” Supportive Care in Cancer 30, no. 1 (2022): 625–634, 10.1007/s00520-021-06435-y. 34355279 PMC8636432

[246] A. M. Henneghan , B. G. Fico , M. L. Wright , S. R. Kesler , and M. L. Harrison , “Effects of Meditation Compared to Music Listening on Biomarkers in Breast Cancer Survivors With Cognitive Complaints: Secondary Outcomes of a Pilot Randomized Control Trial,” Explore (New York, N.Y.) 18, no. 6 (2022): 657–662. 34802955 10.1016/j.explore.2021.10.011 PMC9085959

[247] E. L. Nelson , L. B. Wenzel , K. Osann , et al., “Stress, Immunity, and Cervical Cancer: Biobehavioral Outcomes of a Randomized Clinical Trial [Corrected],” Clinical Cancer Research 14, no. 7 (April 2008): 2111–2118. 18381952 10.1158/1078-0432.CCR-07-1632 PMC4572837

[248] J. L. Steel , D. A. Geller , K. H. Kim , et al., “Web‐Based Collaborative Care Intervention to Manage Cancer‐Related Symptoms in the Palliative Care Setting,” Cancer 122, no. 8 (April 2016): 1270–1282. 26970434 10.1002/cncr.29906 PMC4828258

[249] P. Zhang , L. Mo , X. Li , and Q. Wang , “Psychological Intervention and Its Immune Effect in Cancer Patients,” Medicine (Baltimore) 98, no. 38 (September 2019): e17228. 31567984 10.1097/MD.0000000000017228 PMC6756636

[250] H. X. Wu , H. Zhong , Y. D. Xu , C. P. Xu , Y. Zhang , and W. Zhang , “Psychological and Behavioral Intervention Improves the Quality of Life and Mental Health of Patients Suffering From Differentiated Thyroid Cancer Treated With Postoperative Radioactive Iodine‐131,” Neuropsychiatric Disease and Treatment 2, no. 12 (May 2016): 1055–1060, https://www.ncbi.nlm.nih.gov/pmc/articles/PMC4859420/. 10.2147/NDT.S105460 PMC4859420 27194911

[251] N. Chong Guan , S. Mohamed , L. Kian Tiah , T. Kar Mun , A. H. Sulaiman , and N. Z. Zainal , “Psychotherapy for Cancer Patients: A Systematic Review and Meta‐Analysis,” International Journal of Psychiatry in Medicine 51, no. 5 (July 2016): 414–430, 10.1177/0091217416680197. 28629286

[252] A. M. Barsevick , “A Systematic Qualitative Analysis of Psychoeducational Interventions for Depression in Patients With Cancer,” Oncology Nursing Forum 29, no. 1 (February 2002): 73–87, https://onf.ons.org/onf/29/1/systematic‐qualitative‐analysis‐psychoeducational‐interventions‐depression‐patients‐cancer. 11817494 10.1188/02.ONF.73-87

[253] J. Savard , S. Simard , H. Ivers , and C. M. Morin , “Randomized Study on the Efficacy of Cognitive‐Behavioral Therapy for Insomnia Secondary to Breast Cancer, Part II: Immunologic Effects,” Journal of Clinical Oncology 23, no. 25 (September 2005): 6097–6106. 16135476 10.1200/JCO.2005.12.513

[254] X. Jiang , J. Sun , R. Song , Y. Wang , J. Li , and R. Shi , “Acceptance and Commitment Therapy Reduces Psychological Distress in Patients With Cancer: A Systematic Review and Meta‐Analysis of Randomized Controlled Trials,” Frontiers of Psychology (January 2024): 1–17, 10.3389/fpsyg.2023.1253266/full. PMC10796538 38250124

[255] C. Calderon , M. Gustems , B. Obispo , et al., “The Mediating Role of Exercise in Depression and Fatigue in Patients With Advanced Cancer,” Current Oncology 31, no. 6 (June 2024): 3006–3016, https://www.mdpi.com/1718‐7729/31/6/229. 38920713 10.3390/curroncol31060229 PMC11203259

[256] A. J. Mitchell , N. Meader , E. Davies , et al., “Meta‐Analysis of Screening and Case Finding Tools for Depression in Cancer: Evidence Based Recommendations for Clinical Practice on Behalf of the Depression in Cancer Care Consensus Group,” Journal of Affective Disorders 140, no. 2 (October 2012): 149–160. 22633127 10.1016/j.jad.2011.12.043

[257] M. J. Fisch , J. W. Lee , M. Weiss , et al., “Prospective, Observational Study of Pain and Analgesic Prescribing in Medical Oncology Outpatients With Breast, Colorectal, Lung, or Prostate Cancer,” Journal of Clinical Oncology 30, no. 16 (June 2012): 1980–1988. 22508819 10.1200/JCO.2011.39.2381 PMC3383175

[258] R. Schear , S. G. Eckhardt , E. A. Kvale , R. Richardson , and B. L. Jones , “‘Cancer Life ReiMagined:’ The CaLM Model of Whole‐Person Cancer Care,” JCO 37, no. 27_suppl (Sptember 2019): 74, 10.1200/JCO.2019.37.27_suppl.74.

[259] Y. J. Liu , L. P. Wu , H. Wang , Q. Han , S. N. Wang , and J. Zhang , “The Clinical Effect Evaluation of Multidisciplinary Collaborative Team Combined With Palliative Care Model in Patients With Terminal Cancer: A Randomised Controlled Study,” BMC Palliative Care 22, no. 1 (June 2023): 71. 37312118 10.1186/s12904-023-01192-7 PMC10262549

[260] R. Dantzer , M. W. Meagher , and C. S. Cleeland , “Translational Approaches to Treatment‐Induced Symptoms in Cancer Patients,” Nature Reviews. Clinical Oncology 9, no. 7 (May 2012): 414–426. 10.1038/nrclinonc.2012.88 PMC3412618 22641361

[261] O. Muhorakeye and E. Biracyaza , “Exploring Barriers to Mental Health Services Utilization at Kabutare District Hospital of Rwanda: Perspectives From Patients,” Frontiers in Psychology 12 (2021): 638377. 33828506 10.3389/fpsyg.2021.638377 PMC8019821

[262] H. Mahajan , N. Reddy , N. G. M. Devi , et al., “Projected Cancer Burden, Challenges, and Barriers to Cancer Prevention and Control Activities in the State of Telangana,” PLoS One 18, no. 7 (July 2023): e0278357, 10.1371/journal.pone.0278357. 37450553 PMC10348541

[263] J. Walker , H. Hobbs , M. Wanat , et al., “Implementing Collaborative Care for Major Depression in a Cancer Center: An Observational Study Using Mixed‐Methods,” General Hospital Psychiatry 76 (May 2022): 3–15, https://www.sciencedirect.com/science/article/pii/S0163834322000330. 35305403 10.1016/j.genhosppsych.2022.03.003

[264] A. Duarte , J. Walker , S. Walker , et al., “Cost‐Effectiveness of Integrated Collaborative Care for Comorbid Major Depression in Patients With Cancer,” Journal of Psychosomatic Research 79, no. 6 (December 2015): 465–470. 26652589 10.1016/j.jpsychores.2015.10.012 PMC4678258

[265] K. E. Irwin , E. R. Park , L. E. Fields , et al., “Bridge: Person‐Centered Collaborative Care for Patients With Serious Mental Illness and Cancer,” Oncologist 24, no. 7 (July 2019): 901–910. 30696722 10.1634/theoncologist.2018-0488 PMC6656464

[266] L. A. van Tuijl , M. Basten , K. Y. Pan , et al., “Depression, Anxiety, and the Risk of Cancer: An Individual Participant Data Meta‐Analysis,” Cancer 129, no. 20 (2023): 3287–3299, 10.1002/cncr.34853. 37545248

[267] B. L. Andersen , R. J. DeRubeis , B. S. Berman , et al., “Screening, Assessment, and Care of Anxiety and Depressive Symptoms in Adults With Cancer: An American Society of Clinical Oncology Guideline Adaptation,” Journal of Clinical Oncology 32, no. 15 (May 2014): 1605–1619, https://www.ncbi.nlm.nih.gov/pmc/articles/PMC4090422/. 24733793 10.1200/JCO.2013.52.4611 PMC4090422

